# Anatomical traits related to leaf and branch hydraulic functioning on Amazonian savanna plants

**DOI:** 10.1093/aobpla/plad018

**Published:** 2023-04-24

**Authors:** Priscila F Simioni, Thaise Emilio, André L Giles, Gustavo Viana de Freitas, Rafael Silva Oliveira, Lara Setime, Angela Pierre Vitoria, Saulo Pireda, Ivone Vieira da Silva, Maura Da Cunha

**Affiliations:** Laboratório de Biologia Celular e Tecidual, Universidade Estadual do Norte Fluminense Darcy Ribeiro, Campos dos Goytacazes, RJ, Brasil; Programa Nacional de Pós-Doutorado (PNPD), Programa de Pós-Graduação em Ecologia, Instituto de Biologia, UNICAMP, Campinas, Brasil; Instituo Nacional de Pesquisa da Amazonia (INPA), Manaus, Amazonas, Brasil; Departamento de Fitotecnia, Centro de Ciências Agrárias, Universidade Federal de Santa Catarina, Florianópolis, Brasil; Laboratório de Ciências Ambientais, Universidade Estadual do Norte Fluminense Darcy Ribeiro, Campos dos Goytacazes, RJ, Brasil; Departamento de Biologia Vegetal, Instituto de Biologia, UNICAMP, Campinas, Brasil; Laboratório de Biologia Celular e Tecidual, Universidade Estadual do Norte Fluminense Darcy Ribeiro, Campos dos Goytacazes, RJ, Brasil; Laboratório de Ciências Ambientais, Universidade Estadual do Norte Fluminense Darcy Ribeiro, Campos dos Goytacazes, RJ, Brasil; Laboratório de Biologia Celular e Tecidual, Universidade Estadual do Norte Fluminense Darcy Ribeiro, Campos dos Goytacazes, RJ, Brasil; Laboratório de Biologia Vegetal, Universidade do Estado do Mato Grosso, Alta Floresta, MT, Brasil; Laboratório de Biologia Celular e Tecidual, Universidade Estadual do Norte Fluminense Darcy Ribeiro, Campos dos Goytacazes, RJ, Brasil

**Keywords:** Cerrado, embolism resistance; leaf anatomy, pit membrane thickness; water use efficiency, xylem anatomy

## Abstract

Amazonian savannas are isolated patches of open habitats found within the extensive matrix of Amazonian tropical forests. There remains limited evidence on how Amazonian plants from savannas differ in the traits related to drought resistance and water loss control. Previous studies have reported several xeromorphic characteristics of Amazonian savanna plants at the leaf and branch levels that are linked to soil, solar radiation, rainfall and seasonality. How anatomical features relate to plant hydraulic functioning in this ecosystem is less known and instrumental if we want to accurately model transitions in trait states between alternative vegetation in Amazonia. In this context, we combined studies of anatomical and hydraulic traits to understand the structure–function relationships of leaf and wood xylem in plants of Amazonian savannas. We measured 22 leaf, wood and hydraulic traits, including embolism resistance (as P_50_), Hydraulic Safety Margin (HSM) and isotope-based water use efficiency (WUE), for the seven woody species that account for 75% of the biomass of a typical Amazonian savanna on rocky outcrops in the state of Mato Grosso, Brazil. Few anatomical traits are related to hydraulic traits. Our findings showed wide variation exists among the seven species studied here in resistance to embolism, water use efficiency and structural anatomy, suggesting no unique dominant functional plant strategy to occupy an Amazonian savanna. We found wide variation in resistance to embolism (−1.6 ± 0.1 MPa and −5.0 ± 0.5 MPa) with species that are less efficient in water use (e.g. *Kielmeyera rubriflora*, *Macairea radula*, *Simarouba versicolor*, *Parkia cachimboensis* and *Maprounea guianensis*) showing higher stomatal conductance potential, supporting xylem functioning with leaf succulence and/or safer wood anatomical structures and that species that are more efficient in water use (e.g. *Norantea guianensis* and *Alchornea discolor*) can exhibit riskier hydraulic strategies. Our results provide a deeper understanding of how branch and leaf structural traits combine to allow for different hydraulic strategies among coexisting plants. In Amazonian savannas, this may mean investing in buffering water loss (e.g. succulence) at leaf level or safer structures (e.g. thicker pit membranes) and architectures (e.g. vessel grouping) in their branch xylem.

## Teaser

Our results suggest that species that inhabit hot and seasonal savannas can exhibit different strategies of drought tolerance/avoidance. These strategies can be equally successful in the maintenance of a favourable water balance, allowing species persistence and co-occurrence. Distinct strategies to deal with drought permitted wide divergence regarding embolism vulnerability. Our findings highlight the importance of looking beyond embolism resistance metrics in mega-diverse vegetations to access plant adherence to climate but also drought vulnerability.

## Introduction

The Amazon is well known for its forests but at least 267 000 km^2^ of the area is covered by natural grasslands and savanna-like vegetation (Devecchi *et al*. 2020), grouped under the broader term ‘Amazonian savanna’ (Pires and Prance 1985). Several studies have been performed on the drought resistance of plant species in the Amazonian Forests based on the assessment of their hydraulic traits (Rowland *et al*. 2015; Santiago *et al*. 2018; Barros *et al*. 2019; Bittencourt *et al*. 2020; Fontes *et al*. 2020, Giles *et al.* 2022). In comparison, the knowledge of functional anatomy and hydraulic functioning of savanna species in the Amazon biome is less available. Mechanistic predictions on forest–savanna transitions will benefit from the knowledge of species’ morphological and ecophysiological traits where transitions to open vegetation are likely to occur, as predicted for the Amazon region. For example, we currently know that many Amazonian savanna species show xeromorphic features in their leaf and wood anatomy but not how features related to hydraulic traits related to drought resistance and water loss. This knowledge is necessary if we want to accurately model transitions in trait states between alternative vegetation using Land Surface Models that include recent advances in plant ecophysiology (Eller *et al*. 2020).

Amazonian savannas are isolated patches of open habitats found within the extensive matrix of Amazonian tropical forests. Amazonian savannas differ from other savannas due to their high annual precipitation (~2180 mm) and from nearby Amazonian forests due to their marked seasonality in rainfall and extreme nutrient-poor that prevents forests, favouring savanna-like vegetation to grow instead (Lloyd *et al*. 2015). Previous studies have reported several xeromorphic characteristics of Amazonian Savanna plants at the leaf and branch levels that are linked to soil, solar radiation, rainfall, and seasonality (Simioni *et al*. 2017; Ariano *et al*. 2022). Recent evidence suggests leaf deciduousness coupled with stomatal regulation to be a common water regulation strategy for savanna species in the transition between Amazon and Cerrado biomes (Soares Jancoski *et al*. 2022). This extends previous work on other Neotropical forest–savanna transitions showing higher leaf conductance and capacitance in savanna species but no difference in stem traits, including resistance to embolism (Hao *et al*. 2008). If leaves are key in adapting to savanna-like vegetation, we need to know how different leaf features are related to plant water transport and drought resistance. Currently, several anatomic features have been documented in those vegetations, including leaf anatomy (Simioni *et al*. 2017; Ariano *et al*. 2022), wood anatomy (Simioni *et al*. 2020) and anatomy of secretory structures (Pessoa *et al.* 2021a), but many others like water-use efficiency (WUE), drought-induced resistance to embolism, hydraulic safety margin and ultrastructures of xylem cell types (e.g. pit membrane thickness) are missing. Hydraulic traits, mainly hydraulic safety margin and drought-induced embolism resistance, are critical to assessing drought resistance (Anderegg *et al*. 2016). Furthermore, our acknowledgement studies that relate anatomical and hydraulic traits are missing and necessary if we want to predict plant response to drought while hydraulic traits are still not available for most plants.

Growing knowledge of how anatomical and physiological attributes coordinate to allow plants to adapt to drought shows general trends in the structure of the plants. Different lines of evidence suggest that water loss arises from leaks in two main locations: cuticle and stomata (Richardson *et al*. 2007; Bueno *et al*. 2019). Stomata closure could be delayed in succulent leaves by the supply of water stored in leaf tissues (Rosado and de Mattos 2007; Rosado *et al*. 2016). At the same time, hydraulic functioning is sustained by the stomatal response to leaf turgor guarantees that the plant transpiration rate is proportional to xylem conductivity (Brodribb 2009). In the xylem, lateral flow mediated by the ultrastructural characteristics of the intervascular pits, especially those related to the pit membrane, can also play an important role in preventing the propagation of air bubbles along the vessel network (Choat *et al*. 2008; Lens *et al*. 2011, 2016). This process could explain the direct functional link between membrane thickness and resistance to embolism (Jansen *et al*. 2009; Li *et al*. 2016; Dória *et al*. 2018; Santiago *et al.* 2019).

Evidence from plants growing under seasonal drought conditions, including other savannas, suggests co-variance between vessel attributes and stomatal regulation. For example, previous work relates leaf hydraulics, including turgor loss and leaf conductivity, to wood density in central Brazil savannas (Bucci *et al*. 2004). Less clear is how xeromorphic features relate to physiological traits fundamental to the prediction of drought impacts on forests (Anderegg *et al*. 2016) and savannas (Sankaran 2019). At the leaf level, water restriction and high irradiance reduce isotopic discrimination and increase the amount of ^13^C incorporated in the plant’s leaf tissue as stomata remain closed longer (Martinelli *et al.* 1998; Teixeira *et al*. 2018). From the carbon isotopic ratio (δ^13^C), we can also estimate the WUE (Martinelli *et al.* 1998; Vitória *et al*. 2018), which represents an important integrative trait of functions in the leaf scale. At the branch level, xeromorphic characteristics in the secondary xylem include the presence of a high frequency of vessels, narrower and shorter vessel elements, vasicentric tracheid, thicker fibre walls, thicker intervascular pits, and well-defined growth layers (Carlquist 1977, 2018; Chave *et al*. 2009; Lens *et al*. 2011; Ziemińska *et al*. 2013). Together, the knowledge accumulated in the past decades shows the potential for the coordination of anatomical and hydraulic traits. This has served as the basis for the use of anatomical features as indicators of drought resistance (Dória *et al*. 2018; Levionnois *et al*. 2020). At the same time, evidence of trait co-variation in a single specie under contrasting drought regimes shows that trait–trait correlations change with plant water status (Ramírez-Valiente *et al*. 2020). This suggests that trait–trait relationships are context dependent and assuming trait correlations without prior knowledge of those relationships under similar conditions may be misleading.

In this context, we combined studies of anatomical and hydraulic traits to understand the structure–function relationships of leaf and xylem in plants of Amazonian savannas. We measured 22 leaf, wood, and hydraulic traits of seven dominant woody species, making this the most comprehensive study to date on anatomical traits (including leaf, wood and ultrastructure features) and the first estimate of drought-induced xylem embolism resistance of Amazonian savanna plants.

To understand trait–trait relationships of Amazonian savanna plants, we asked:

(i) What are the dominant structural and hydraulic traits?
(ii) How hydraulic and anatomic traits are related to each other?
(iii) Which anatomical traits are more strongly related to hydraulic traits?

**Figure 1.**
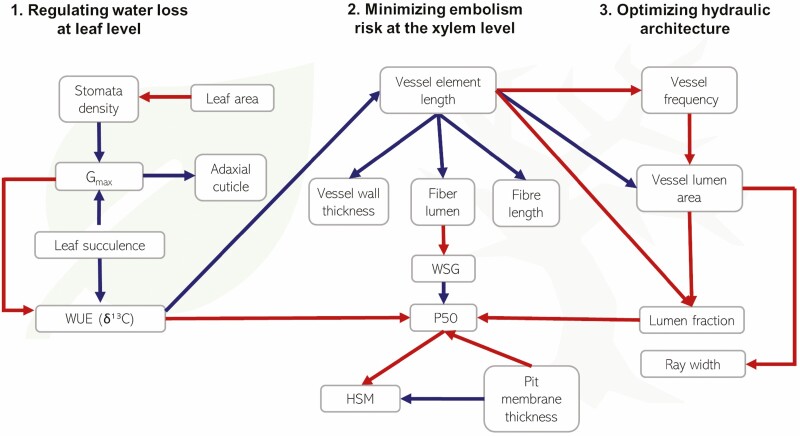
Different ways in which plants may adjust their leaf and wood anatomy to cope with drought. (1) *Regulating water loss at leaf level*. Abundant stomata and thicker cuticles allow for high transpiration and water loss control while tissue succulence sustains transpiration flow at leaf level; (2) *minimizing embolism risk at stem level.* A safe wood structure results from short, reinforced vessels with thicker pit membranes; (3) *optimizing hydraulic architecture to sustain transpiration demand.* Water supply is guaranteed by trade-offs in vessel length, frequency and lumen area. Blue arrows depict positive relationships and red arrows negative ones. This theoretical model was constructed mostly based on references provides in the main text, particularly by Hacke *et al.* (2001), Richardson *et al.* (2007), Jansen *et al.* (2009), Zanne *et al.* (2010); Lens *et al.* (2011), Ziemińska *et al.* (2013), Hacke (2015), de Boer *et al.* (2016), Rosado *et al.* (2016), Carlquist (2018), Dória *et al.* (2018), Teixeira *et al.* (2018), Bueno *et al.* (2019).

We hypothesize that in highly diverse ecosystems, such as Amazonian savannas, trees may adjust their wood and leaf anatomy in different ways to adapt to drought (cf. Fig. 1 for details). This will result in the coexistence of several contrasting functional strategies coexisting under the same environmental conditions (Rosado *et al*. 2013; Dias *et al*. 2020). As regulation of water loss is expected to be the main selective force in high-radiation and high-seasonality environments, we expect different alternative functional designs based on strong or weak regulation of water loss by plants. In plants with strong control of water loss (i.e. high WUE), we expect to observe high-resistance to embolism and reinforcement of anatomical structures (e.g. thicker vessel walls) to resist high xylem tension. In plants with weak control of water loss (i.e. low WUE), we expect to observe either low-resistance to embolism or xylem architecture adjustments that minimize embolism formation.

## Materials and Methods

### Study area

**Figure 2.**
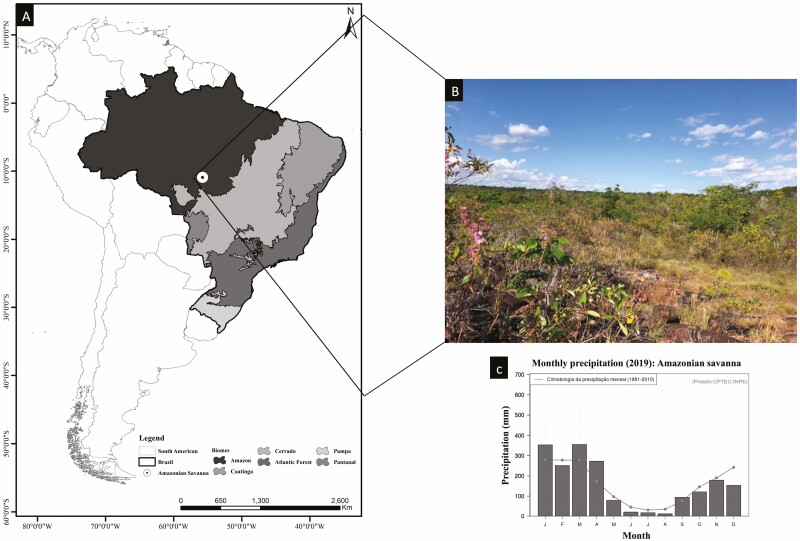
Study region, in North Brazil: (A) Map of the study region, in North Brazil, showing the specific location; (B) Amazonian savanna landscape with vegetation occurring in rocky outcrop seasonally dry environments; (C) climate graph with average rainfall for 2019 (Source: INPE).

The study was conducted in a rocky Amazonian savanna community in the municipality of Nova Canaã do Norte in the state of Mato Grosso, Brazil (Fig. 2), 10°53ʹ98,7″ S, 55°46ʹ68,7″ W. The soil of the study area is classified as Litholic Neosols that are poorly drained, dystrophic, alic, extremely acidic and sandy, and with low-nutrient concentrations (Pessoa *et al.* 2021b). The climate of the area is equatorial (Am) hot and humid, according to the Köppen classification, with temperatures varying from 20 °C to 36 °C with an annual average above 28 °C (Alvares *et al*. 2013). Total annual rainfall can reach 2180 mm, with two well-defined seasons [see Supporting Information—Figure S5]—a rainy season encompassing November, December, January, February and March (1180 mm) and a dry season encompassing June, July, August and September (108 mm)—with the other months being considered transition periods (Alvares *et al*. 2013). Plants growing in savannas under that conditions are expected to be subjected to more intensive droughts than in typical oxisol savannas plants due to their limited groundwater, an example of other rock outcrops in the tropics (Brum *et al*. 2017).

### Data collection

The dominant woody plant species in the study area accordingly to a previous study (Pessoa *et al.* 2021b) were selected for this study. We focussed our measurements on the seven species most abundant species that together represent 75% of the community plant biomass (Table 1). The following morphological, anatomical and hydraulic measurements were made for each species: leaf area, leaf succulence, leaf specific mass, stomatal density, adaxial cuticle thickness, maximum theoretical stomatal conductance and carbon isotopic compositionWUE, theoretical hydraulic conductivity, vessel lumen area, vessel frequency, vessel element length (including the vessel element tails **[see Supporting Information—Figure S1]**), lumen fraction, ray frequency and ray width, fibre lumen, fibre length, wood-specific gravity, vessel wall thickness, intervessel pits (often horizontal in tangential sections **[see Supporting Information—Figure S2]**) and pit membrane thickness (resistance mechanism), and xylem embolism resistance (P50), and hydraulic safety margin (HSM). Trait sampling and measurements for the species are detailed later.

**Table 1.** List of the seven most abundant species in the rocky outcrop Amazonian savanna community, in the town of Nova Canaã do Norte, Mato Grosso, Brazil. PI, proportion of individuals in relation to the total plot area (764 individuals per ha^−1^; 75% of the plot species were analyzed).

Family	Species	Habit	Leaf habit	PI, %
Calophyllaceae	*Kielmeyera rubriflora* Cambess.	Shrub/tree	DE^1^	21.35
Fabaceae	*Parkia cachimboensis* H.C.Hopkins	Tree	EV^*^	16.50
Melastomataceae	*Macairea radula* (Bonpl.) DC.	Shrub/tree	EV^2^	12.30
Euphorbiaceae	*Alchornea discolor* Poepp.	Shrub/tree	DE^1^	7.70
Marcgraviaceae	*Norantea guianensis* Aubl.	Shrub/tree	EV^1^	7.70
Simaroubaceae	*Simarouba versicolor* A.St.-Hil	Tree	SD/EV^1^	5
Euphorbiaceae	*Maprounea guianensis* Aubl.	Tree	EV^1^	4.45

^1^ Kattge *et al.* (2011) and

^2^ Somavilla and Graciano-Ribeiro (2011).

^*^No information available from literature but not seen without leaves in our field studies.

EV: evergreen; DE: decidual; SD: semi-decidual.

### Leaf allometry and anatomy

Leaf area was determined by digitally scanning the entire leave (subsequently used for the other morphological measurements) and then measuring their area using ImageJ digital image processing software.

Morphological measurements were made of samples from five fully expanded leaves from three individuals of each species. Discs of 0.5 cm in diameter were removed from the median third of the leaves. The discs were hydrated for 24 h and dried with paper for subsequent determination of saturated mass (*M*_saturated_) using a digital balance (AY220, Shimadzu), and thickness with a digital calliper (Stainless, Hardened). The hydrated discs were then placed in an oven set at 55 °C for 72 h to obtain dry mass (*M*_dry_). These parameters were used to calculate leaf succulence (Kluge and Ting 1978), according to the following equation:


$$\begin{array}{l} LS=\left(M_{saturated}-\frac{M_{dry}}{Area_{disc}}\right) \end{array}$$ (1)

and leaf mass per area (Kluge and Ting 1978), according to the following equation:


$$\begin{array}{l} LMA=\left(\frac{M_{dry}}{Area_{disc}}\right) \end{array}$$ (2)

Leaf cross-sections were made in the middle of the leaf by freehand for measurements of leaf anatomical attributes and to observe the adaxial cuticle. Stomatal density (mm^2^) and stomatal pore length (µm) were determined from the analysis of images of the epidermis dissociated by the Franklin method (Franklin 1945). Theoretical maximum stomatal conductance was calculated from the relationship between stomatal pore density and size (de Boer *et al*. 2016), according to the following equation:


$$\begin{array}{l} G_{\max}=\;D\;\times \;L \end{array}$$ (3)

where *G*_max_ is the theoretical maximum stomatal conductance, *D* is stomatal density (mm²) and *L* is stomatal length (µm).

Two leaves were selected for each species for investigation of stomatal morphology on the abaxial leaf face using scanning electron microscopy techniques. Fragments of the median third of the leaf blade were fixed in an aqueous solution of 2.5% glutaraldehyde, 4% formaldehyde and 0.05 M sodium cacodylate buffer at pH 7.2 (Karnovsky 1965 modified by Da Cunha *et al*. 2000) and then post-fixed in 1% osmium tetroxide and 0.05 M sodium cacodylate buffer for 2 h at room temperature. After fixation, the samples were submitted to acetone dehydration, followed by CO_2_ critical point drying (CPD 030, Baltec). The samples were then adhered to stubs with carbon tape and covered with a layer of ~20 nm of gold (SCD 050, Baltec, Switzerland). Images were obtained using a ZEISS EVO 40 (Germany) scanning electron microscope at a voltage of 15 kV.

### Branch allometry and anatomy

Branch samples were sectioned (15–20 μm thickness) in transversal and longitudinal tangential planes using a sliding microtome (SM2010 R, LEICA, Germany). The sections were then clarified in sodium hypochlorite (50%) and acidulated water (0.1%), dehydrated in an ascending ethanol series (50–100%) (Johansen, 1940), stained with Astra blue and hydro-alcoholic Safranin, and immersed in xylene P.A. Permanent slides were made using Entellan (Merck) synthetic resin.

We used a maceration method for the measurements of vessel element length (including tips) and fibre length. Maceration of branch material followed Franklin (1945). Small branch fragments were removed from each sample and placed in bottles containing a macerating solution of glacial acetic acid and hydrogen peroxide (1:1). The bottles were then sealed and placed in an oven at 60 °C for 24 h or until the complete dissociation of cells. The material was then washed in distilled water, stained with 1% aqueous Safranin, and mounted on semi-permanent slides with glycerine. Imperforate tracheary elements were not observed in macerations for any of our samples, as it is common among Angiosperm species.

Quantitative analysis was performed using 12 slides per individual. All descriptions, counts and branch cellular measurements followed IAWA Committee standards (1989). Permanent and semi-permanent slides were analyzed using a light-field light microscope (Axioplan, ZEISS, USA), with image capture via a coupled camera (Power shot A640, CANON, USA).

Two branches of each species were selected for analysis of the intervascular pit membrane using transmission electron microscopy. Branch fragments (ca. 1 cm) were fixed in a modified Karnovsky solution (Karnovsky 1965 modified by Da Cunha *et al*. 2000). Post-fixed in 1% osmium tetroxide and 0.05 M sodium cacodylate buffer for 2 h at room temperature, and then dehydrated in an increasing acetone series and infiltrated and embedded with epoxy resin (Epon). Ultrathin sections (80 nm) were made using an ultramicrotome (Reichert Ultracuts Leica Instruments) with a diamond knife (Diatome), which were collected in copper grids (300 mesh) and contrasted with 1.0% alcoholic uranyl acetate, followed by 5.0% aqueous lead citrate (Reynolds, 1963). Ultrastructure analysis of pit membranes was performed using a JEM 1400 Plus JEOL transmission electron microscope at a voltage of 80 kV, with 20 observations per individual. Measurements of anatomical attributes (Table 2) were performed using the Image Pro-Plus 4.0 digital image processing system. Pit membrane thickness was measured by cuts made in the sapwood at three different points, later averaged to represent the sample pit membrane thickness **[see Supporting Information—Figure S4]**. All measurements for each species were made from three individuals with similar heights and two branches between 1.5 and 2 cm in diameter per individual.

**Table 2.** Mean of traits measured in each dominant species of the Amazonian savanna (mean ± standard deviation).

Traits	Abbreviation	Units	*Kielmeyera rubriflora*	*Parkia cachimboensis*	*Macairea radula*	*Simarouba versicolor*	*Maprounea guianensis*	*Alchornea discolor*	*Norantea guianensis*
Leaf area	LA	cm²	16.99 ± 1.20	38.26 ± 3.17	16.33 ± 0.31	17.65 ± 1.31	18.21 ± 2.12	31.18 ± 4.99	58.79 ± 3.81
Leaf succulence	LS	g.m^−2^	126.45 ± 28.17	117.48 ± 22.25	279.31 ± 14.58	177.64 ± 8.84	78.56 ± 10.89	54.96 ± 4.08	151.66 ± 6.83
Leaf mass per area	LMA	g.m^−2^	59.21 ± 13.17	67.00 ± 14.05	102.85 ± 17.71	116.07 ± 22.17	59.45 ± 9.37	78.79 ± 8.85	120.31 ± 11.70
Stomatal density	SD	mm²	27.59 ± 0.68	25.36 ± 2.89	34.93 ± 4.62	25.40 ± 0.40	28.47 ± 2.04	10.40 ± 0.72	10.27 ± 0.64
Adaxial cuticle	AC	µm	1.81 ± 0.27	2.84 ± 0.67	8.13 ± 3.38	3.10 ± 0.12	7.07 ± 2.11	8.22 ± 1.38	4.93 ± 0.22
*G*_max_	*G*_max_		165.03 ± 11.83	197.73 ± 3.90	276.56 ± 34.61	241.85 ± 16.34	173.47 ± 5.70	101.12 ± 4.74	114.87 ± 4.49
*K*_h_	*K*_h_	kg s^−1^ m^−1^ MPa^−1^	2.54E+09 ± 6.16E+08	3.90E+09 ± 1.60E+09	6.48E+08 ± 5.30E+07	4.53E+09 ± 1.38E+09	1.67E+09 ± 8.00E+08	7.10E+09 ± 2.89E+09	4.28E+09 ± 1.89E+09
Vessel lumen area	VLA	µm	3489.52 ± 443.91	3455.80 ± 490.09	2450.62 ± 472.66	3716.75 ± 781.53	2398.84 ± 275.69	6636.97 ± 1139.66	4914.62 ± 293.20
Frequency vessel	FV	mm²	44.31 ± 16.79	57.83 ± 6.67	93.47 ± 12.78	74.68 ± 18.00	49.79 ± 9.53	30.12 ± 10.32	39.93 ± 12.09
Vessel element length	VL	µm	418.48 ± 38.54	205.28 ± 4.17	360.13 ± 35.27	311.55 ± 16.80	461.53 ± 52.60	658.23 ± 106.32	714.85 ± 78.65
Lumen fraction	LF		0.24 ± 0.02	0.35 ± 0.07	0.30 ± 0.03	0.37 ± 0.04	0.27 ± 0.01	0.18 ± 0.02	0.21 ± 0.03
Ray frequency	RF	mm	8.70 ± 0.79	10.14 ± 0.57	10.26 ± 1.41	7.93 ± 1.02	14.09 ± 0.43	11.05 ± 0.36	4.79 ± 1.19
Ray width	RW	µm	19.78 ± 0.80	14.79 ± 3.32	12.18 ± 2.50	35.64 ± 1.80	14.95 ± 2.82	22.91 ± 1.10	84.24 ± 9.09
Fibre length	FLE	µm	654.45 ± 60.22	647.53 ± 51.42	456.38 ± 20.26	540.74 ± 15.71	723.51 ± 58.11	1095.58 ± 108.46	1005.62 ± 86.88
Fibre lumen	FLU	µm	7.72 ± 0.64	7.17 ± 1.19	7.21 ± 1.18	7.77 ± 0.51	8.31 ± 0.34	14.92 ± 1.17	16.67 ± 0.81
Wood specific gravity	WSG	g.cm^−3^	0.57 ± 0.03	0.49 ± 0.04	0.63 ± 0.01	0.51 ± 0.04	0.48 ± 0.01	0.50 ± 0.04	0.46 ± 0.02
Vessel wall thickness	VWT	µm	2.84 ± 0.26	3.46 ± 0.55	2.52 ± 0.39	2.56 ± 0.18	3.81 ± 0.23	5.23 ± 0.66	3.95 ± 0.25
Intervessel pits	IPit	µm	8.77 ± 1.20	6.48 ± 0.79	5.66 ± 0.16	5.57 ± 0.17	4.41 ± 0.34	11.36 ± 0.65	8.47 ± 0.29
Thickness pit membrane	TPM	µm	0.31 ± 0.04	0.39 ± 0.04	0.33 ± 0.02	0.32 ± 0.02	0.41 ± 0.04	0.33 ± 0.01	0.27 ± 0.006
WUE (δ¹³C)	WUE	‰	−28.80 ± 0.10	−30.07 ± 0.65	−29.5 ± 0.17	−30.77 ± 0.71	−28.43 ± 0.21	−28.53 ± 0.21	−27.20 ± 0.10
P_50_	P_50_	MPa	−3.8 ± 0.08	−5.0 ± 0.46	NA	−4,4 ± 0,32	−4,5 ± 0,92	NA	−1.6 ± 0.06
HSM	HSM	MPa	2.8 ± 0.03	3.8 ± 0.19	NA	2,6 ± 0,19	3,3 ± 0,97	NA	0.4 ± 0.12

Wood-specific gravity was calculated from two branches per individual by first measuring the fresh volume of wood samples by displacement of a water column (Williamson and Wiemann 2010). Samples were immersed in a beaker containing water on top of a digital balance, and sample volume was converted from the weight of displaced water (e.g. 1 g = 1 cm^3^). Dry mass was obtained by drying the samples in an oven at 105 °C for 72 h. Wood-specific gravity was then calculated as


$$\begin{array}{l} WSG=\frac{D_{m}}{D_{v}} \end{array}$$ (4)

where WSG is the wood-specific gravity (g cm^−3^), *D*_m_ is the dry mass and *D*_v_ is the displaced volume.

Theoretical hydraulic conductivity (*K*_h_) was calculated from the lumen diameter of 30 randomly selected vessels for each sampled individual using the Hagen–Poiseuille equation:


$$\begin{array}{l} K_{h}=\frac{\pi D4}{128\eta } \end{array}$$ (5)

where *K*_h_ is the theoretical hydraulic conductivity in kg s^−1^ m^−1^ MPa^−1^, η is the water viscosity at 20 °C (1.002 × 10^−9^ MPa s) and *D* is the hydraulically weighted vessel diameter in mm.

Because cross-sections of vessels are not perfect circles, vessel lumen area was used to calculate equivalent vessel diameter (*d*) (Scholz *et al*. 2013) as


$$\begin{array}{l} d=\surd 4A.\pi \end{array}$$ (6)

where *A* is the vessel lumen area.

Hydraulically weighted vessel diameter (*D*) was calculated as


$$\begin{array}{l} D={\left(\frac{\Sigma d^{4}}{N}\right)}^{0.25} \end{array}$$ (7)

where *d* is the equivalent vessel diameter in mm and *N* is the number of measured vessels.

### Hydraulic traits

*Carbon isotopic composition (δ* ^*13*^ *C)*. For determining δ^13^C, five leaves were selected from three individuals of each species. The leaves were dried in an oven at 60 °C for 72 h and then macerated. After maceration, the five leaves for each individual were homogenized. The homogenized material was subsequently weighed (1.5 mg) with a precision analytical balance. Data were obtained using a Thermo Finnigan Delta V Advantage mass spectrometer coupled to a Flash 2000 (Thermo 26 Fisher Scientific in Bremen, Germany) elemental analyzer at the Laboratório de Ciências Ambientais from Universidade Estadual do Norte Fluminense Darcy Ribeiro. Pee Dee Belemnite (PDB) was used as the standard value for C. The analytical precision was ±0.1 ‰, while the precision of the elemental and isotopic compositions was determined by certified standard (Protein OAS/IsotopeCert 114859; Elemental Microanalysis).

*Embolism resistance (P*_*50*_). Two branches of 1.5–2.0 m in length per individual (six per species) were collected at dawn for assessing embolism resistance and hydraulic safety (P_50_ and HSM). Long segments of 10–15 cm were cut from the base of each branch under water and were allowed to rehydrate for 12 h, keeping them covered and sealed by black plastic bags. We started measuring the water potential of the leaves after 12 h of hydration, taken to represent the timepoint when transpiration is at its minimum, and the water potential of the plant is closest to equilibrium with that of the soil. Hydraulic measurements were then made on the distal end of each branch to ensure there were no artificially embolized vessels in the measured sample. All samples used for hydraulic measurements were from first- or second-order branches that were 30–55 cm in length and 2–4 cm in diameter and were cut under water with a sharp blade before connecting to the apparatus to ensure that all vessels were open. P_50_ was used as an embolism resistance index, which is water potential corresponding to a 50% loss of xylem conductivity. Xylem embolism resistance of each branch was measured using the pneumatic method in the manual measurements set-up (Pereira *et al*. 2016; Zhang *et al*. 2018). With this method, loss of hydraulic conductance is estimated from the increase in air volume inside the wood caused by the formation of an embolism, as the branch dehydrates (for details of the methods, see Bittencourt *et al*. 2018). Branches were dehydrated using the bench dehydration method (Sperry *et al*. 1988). The branches were bagged for an hour to balance the water potential of the xylem with that of the leaves before each air removal measurement. The volume of the air reservoir was adjusted when a rapid drop in the air discharge or values close to atmospheric pressure was detected to preserve the accuracy of the method. The reservoir volume varied between 1.305 and 2.610 depending on the species and/or individuals in the volume. The water potential of one or two leaves was measured immediately after air removal. Embolism resistance is then given by increasing air removal (PAD = the percentage of air removed) with each tree xylem water potential. To calculate P_50_, we gathered data for the repetitions of two branches of the same tree and adjusted a sigmoid curve to the data, where P_50_ and slope are the adjusted parameters (Pammenter and Willigen 1998).


$$\begin{array}{l} PAD=100/(1+\exp(a\left(\Psi P_{50}\right)\theta \end{array}$$ (8)

Characterization of P_50_ was done for the seven dominant species of the community. The pneumatic method was applied to the branches to construct vulnerability curves for the xylem. We were not able to produce reliable vulnerability curves for *Macareia radula* and *Alchornea discolor* using the pneumatic method most likely due to an undetected leakage during the measurements in the field. After inspecting the curves, we observed a large amount of variation in the initial and final measurements, and consequently, a poor fit of the curves. Both species have very thick bark and fast shrinkage requiring fitting adjustments during the measurements. We believe that this may have caused air leakage and for this reason, we were not confident to use the data collected for both species for the analysis. The P_50_ and HSM are presented for the remaining five dominant species: *N. guianensis*, *S. versicolor*, *P. cachimboensis*, *K. Rubriflora*, and *M. guianensis***[see Supporting Information—Figure S6]**.

*Hydraulic safety margin (HSM) –* HSM is a good predictor of drought resistance (Barros *et al*. 2019). It was calculated from the difference between P_50_ and midday water potential (Ψ_md_), which represents the minimum Ψ of the plant in the dry season **[see Supporting Information—Figure S5]**. This measure is affected by any cuticular or stomatal transpiration and, thus, broadly captures the integrated effects of plant traits and the environmental water demand on the minimum water potential a plant reaches in natural conditions. Leaf water potentials were measured using a pressure chamber (Model 1505, PMS).

### Statistical analysis

To identify the dominant structural and hydraulic traits, how they are related to each other and, particularly, how anatomical traits relate to hydraulic traits, we did the following. First, we examine the correlations between all traits to identify the traits that are more strongly correlated with each other. A correlation matrix was constructed to observe associations among the variables of this study. Correlations were performed on individual observations (*N* = 14) using non-parametric statistics to calculate Spearman’s Coefficient (rho), as data and variables did not follow a normal distribution. Significant correlations among traits (i.e. with a *P* value below the threshold of 0.05) were graphically represented using trait co-variation networks using the R package qgraph (Epskamp *et al*. 2012). Trait correlations networks here represents Spearman’s correlations between variables as weighted edges, where zero indicates no correlation and negative values are comparable in weight to positive values. Then, we extract the main axis of trait co-variation by reducing the dimensionality of our dataset with Principal Component Analysis (PCA) from the vegan package (Oksanen *et al.* 2022) and identify the traits related to the variation on these axes by correlating the PCA loadings against each trait. To assess the relative importance of variation within species in relation to variation among species, we performed a permutational multivariate analysis of variance (permANOVA) from a Euclidian distance matrix (Anderson 2001) calculated over trait values. Finally, we analyze how hydraulic traits relate to anatomical traits using generalized linear mixed models that account for species relatedness and compare the strength of observed relationships using model effect sizes.

Generalized linear mixed-effects random intercept models were fit in the package lme4 (Bates *et al*. 2015) between WUE (*N* = 21), P_50_ (*N* = 14), and HSM (*N* = 14) as dependent and all 19 anatomical traits as independent variables with species-nested-within-genus and genus-nested-within-family (family|genus|species) to account for within-species correlations and the phylogenetic structure of the data. As all random intercept models were significant, we adopt this model and reported the results of linear models in the **supporting information** for comparison (**[see Supporting Information—Figure S7]** and **[see Supporting Information—Table S2]**). To quantify the model goodness of fit, we considered the marginal and conditional *R*^2^. The marginal *R*^2^ indicates how much of the model variance is explained by the fixed effects only, whereas the conditional *R*^2^ indicates how much of the model variance is explained by the complete model, fixed, and random effects. For model comparisons, we reported standardized effect sizes (model slopes) for all models following the recommendation from Nakagawa (2004). Standardized regression coefficients were plotted for each model associated with a 95% confidence interval and compared against each other. This allowed for the evaluation of the significance and strength of the relationship between hydraulic traits (WUE, P_50_, and HSM) and the 19 anatomical features measured. All descriptive and statistical analyses were performed using R software (R Core Team 2022).

## Results

### Trait variation

**Figure 3.**
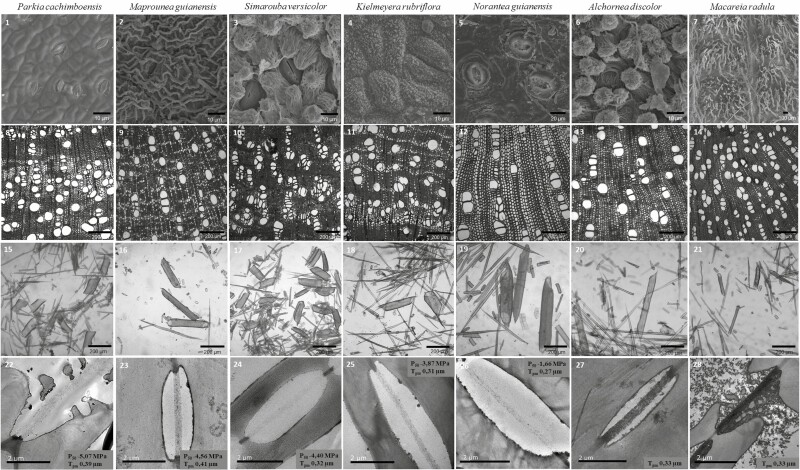
Leaf and wood anatomy of each dominant species in the studied Amazonian savanna. Images 1 to 7: scanning electron microscopy showing different sizes of stomatal pores and layers of epicuticular wax. Images 8 to 14: light microscopy showing the different frequencies of vessels, lumen area, and lumen fractions by area. Images 15 to 21: light microscopy showing the different lengths of vessel elements. Images 22 to 28: transmission electron microscopy showing pit membrane thickness.

The dominant woody species in the studied Amazonian savanna show a wide variation in their anatomical features (Fig. 3) and hydraulic traits of leaf and wood (Table 2). The variation from observations by a factor of two to three was observed among the seven species for most traits, including leaf area (16.99 to 58.79 cm²), vessel frequency (30.12 to 93.47 mm²), lumen area (2450.62 to 6636.97 µm), lumen fraction (0.18 to 0.37), intervascular wall thickness (2.52 to 5.23 µm), fibre length (456.38 to 1095.58 µm), P50 (−1.6 to −5.0 MPa), stomatal density (10.27 to 34.93 mm²) and G_max_ (101.12 to 276.56). This observation was even greater, five times or more, for the attributes of succulence (54.96 to 279.31 g.m^−2^) and HSM (0.4 to 3.8 MPa). Exceptions to this pattern were the traits of wood density (0.46 to 0.63 g.cm^−3^), WUE (−27.20 to −30.77 ‰), and pit membrane thickness (0.27 to 0.41 µm), which varied from 10–30% among species. It is worth mentioning that the relative ordering of species varied according to attributes **[see Supporting Information—Figure S8]**, with no species having a consistent pattern of co-variation for several attributes simultaneously.

Differences in functional strategies among species are noticeable from how average trait values are distributed across species. It was not possible to identify a single hydraulic strategy that converged among different species, on the contrary, species show very distinct patterns of trait value distribution. Among all species, *M. radula* was the species where extreme trait values were prevalent. From the 22 hydraulic and anatomic traits measured, this species presented extreme values more than half of them (*N* = 13). At the leaf scale, this includes the highest values of leaf succulence (slope: 279.31 ± 14.58), stomatal density (slope: 34.93 ± 4.6), adaxial cuticle (slope: 8.13 ± 3.38), G_max_ (slope: 276.56 ± 34.61), and the lowest value of leaf area (slope: 16.33 ± 0.31). At the same time, these species show the lowest trait values for many of the xylem traits, including K_h_ (slope: 6.48E+08 ± 5.30E+07), vessel lumen area (slope: 2450.62 ± 472.66), ray width (slope: 12.18 ± 2.50), fibre length (slope: 456.38 ± 20.26), fibre lumen (slope: 7.21 ± 1.18), vessel wall thickness (slope: 2.52 ± 0.39), the highest trait values for frequency of vessels (slope: 93.47 ± 12.78), and wood density (slope: 0.63 ± 0.01). This pattern almost mirrors the trait value patterns presented by *A. discolor* where half of the trait values were also extreme but in opposite directions. For example, *A. discolor* show the lowest trait values for leaf succulence (slope: 54.96 ± 4.08), stomatal density (slope: 10.40 ± 0.72) and *G*_max_ (slope: 101.12 ± 4.74), where *M. radula* scored the highest values while presenting the highest trait value for many of the same xylem traits which *M. radula* show the lowest, including *K*_h_ (slope: 7.10E+09 ± 2.89E+09), vessel lumen area (slope: 6636.97 ± 1139.66), fibre length (slope: 1095.58 ± 108.46)and fibre lumen (slope: 14.92 ± 1.17).

In contrast, other species show much less extreme values. For example, *P. cachimboensis* and *S. versicolor* have only four and five extreme values, respectively. This includes the shortest vessel element (slope: 205.28 ± 4.17) and the narrowest fibre lumen (slope: 7.17 ± 1.19) for *P. cachimboensis*; and for *S. versicolor*, the largest lumen fraction (slope: 0.37 ± 0.04) and the thinnest vessel walls (slope: 2.56 ± 0.18). Interestingly, those species that are intermediary in most morphological traits show extreme values of hydraulic traits with *P. cachimboensis* showing the highest embolism resistance (slope: −5.0 ± 0.46) and the wider hydraulic safety margin (slope: 3.8 ± 0.19) and *S. versicolor* showing the lowest WUE (slope: −30.77 ± 0.71). *M. guianensis* is another species where only a few (five out of 22) traits show extreme values. This includes the smaller leaf mass per area (slope: 59.45 ± 9.37), the widest lumen vessel area (slope: 2398.84 ± 275.69), the lowest ray frequency (slope: 14.09 ± 0.43), the thinnest intervessel pits (slope: 4.41 ± 0.34) and the thickest pit membranes (slope: 0.41 ± 0.04). Although values for hydraulic traits were not extreme for this species, they were numerically very close to observed values observed for species with extreme values of hydraulic traits. Extreme values of hydraulic traits, however, can also be shown by species with extreme values for morphological traits. Here, one species, *N. guianensi*s, presented the highest WUE (slope: −27.20 ± 0.10), the lowest embolism resistance (slope: −1.6 ± 0.06) and the narrower hydraulic safety margin (slope:0.4 ± 0.12) at the same time that present half of the traits measures as the highest or lowest values in the range. Specifically, this species shows the largest leaf area (slope: 58.79 ± 3.81), the highest leaf mass per area (slope: 120.31 ± 11.70), the lowest stomatal density (slope: 4.93 ± 0.22), the longest vessel element length (slope: 714.85 ± 78.65), the lowest vessel element frequency (slope: 39.93 ± 12.09), the wider ray width (slope: 84.24 ± 9.09), the larger fibre lumen (slope: 16.67 ± 0.81), the lower wood density (slope: 0.46 ± 0.02) and the thinner pit membranes (slope: 0.27 ± 0.006). Intriguingly, *K. rubriflora* presented intermediary values for all traits but leaf mass per area (slope: 59.21 ± 13.17) and adaxial cuticle (slope: 1.81 ± 0.27) that were, respectively, the smallest and the thinner among all species.

### Trait covariation

**Figure 4.**
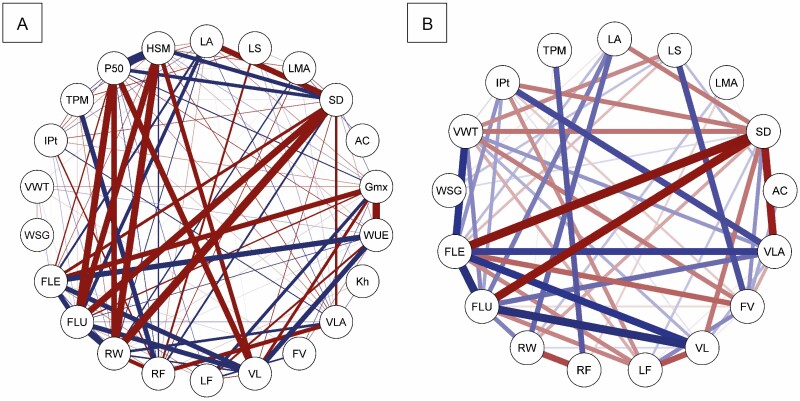
Spearman’s correlation network between (A) anatomical traits and (B) anatomical and hydraulic traits. This represents correlations between variables as weighted edges, where zero indicates no correlation and negative values are comparable in weight to positive values. Positive correlations are denoted in blue tones and negative correlations are in red tones. Correlation strengths are represented by line thickness. Only correlations with a *P* value below the threshold of 0.05 are shown. LA: leaf area; LS: leaf succulence; LMA: leaf mass per area; SD: stomatal density; AC: adaxial cuticle; *G*_max_ (Gmx): theoretical maximum stomatal conductance; WUE (δ¹³C): water-use efficiency; *K*_h_: theoretical hydraulic conductivity; VLA: vessel lumen area; FV: frequency vessel; VL: vessel element length; LF: lumen fraction; RF: ray frequency; RW: ray width; FLU: fibre lumen; FLE: fibre length; WSG: wood-specific gravity; VWT: vessel wall thickness; IPit: intervessel pits; TPM: thickness pit membrane; P50: embolism resistance; HSM: hydraulic safety.

Anatomical traits were strongly correlated to each other (Fig. 4A) with each trait correlated to at least two other traits and moderately connected to many others. Correlations higher than 0.5 were observed in 97 out of 171 pairwise correlations between anatomical traits **[see Supporting Information—Figure S9]**. Among those traits, the fibre length, vessel element length, vessel lumen area, fibre lumen, intervessel lumen, intervessel pits, vessel wall thickness, ray width, stomata density, frequency of vessels, lumen fraction and *G*_max_ and were moderate to strong (*r* > 0.5) correlated to at least other 10 anatomical traits. Strong correlations (*r* > 0.8) were observed mostly between xylem anatomical features and *G*_max_. *G*_max_ was strongly correlated to lumen fraction (*r* = 0.88), frequency of vessels (*r* = 0.84), and fibre length (*r* = −0.91). Lumen fraction was also strongly correlated to the frequency of the vessel (*r* = 0.81) and vessel element length (*r* = −0.82). Vessel element length in turn was strongly correlated to vessel lumen area (*r* = 0.83) and vessel lumen area strongly correlated to *K*_h_ (*r* = 0.82) and intervessel pits (*r* = 0.81). However, fewer anatomical traits were strongly related to hydraulic traits (Fig. 4B). Strong correlations were only observed among WUE and vessel element length (*r* = 0.9) and hydraulic safety margin and ray width (*r* = −0.84). Moderate correlations were more common and significant for nearly all trait combinations **[see Supporting Information—Figure S9]**, but they were visibly stronger for anatomical traits that are highly correlated to each other such as vessel element length, fibre lumen and length.

**Figure 5.**
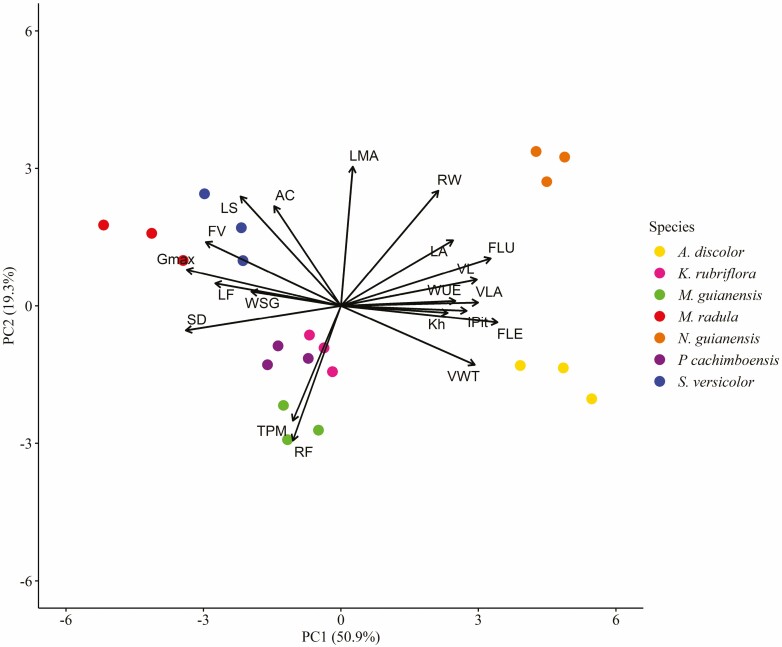
Principal component analysis (PCA) of anatomical and hydraulic traits with species coded by colours. LA: leaf area; LS: leaf succulence; LMA: leaf mass per area; SD: stomatal density; AC: adaxial cuticle; G_max_ (Gmx): theoretical maximum stomatal conductance; WUE (δ¹³C): water-use efficiency; Kh: theoretical hydraulic conductivity; VLA: vessel lumen area; FV: frequency vessel; VL: vessel element length; LF: lumen fraction; RF: ray frequency; RW: ray width; FLU: fibre lumen; FLE: fibre length; WSG: wood-specific gravity; VWT: vessel wall thickness; IPit: intervessel pits; TPM: thickness pit membrane.

The variation from individual trait observation could be summarized in two dominant axes that represent nearly 70% of trait co-variation. The PCA including anatomical and hydraulic (WUE (δ¹³C)) traits explained 70.2 % of the total variation with the first two axes (Fig. 5). The first axis explained 50.9 % of the total variation. This axis was strongly correlated to stomatal density (*r* = −0.94), *G*_max_ (*r* = −0.94), fibre lumen (*r* = 0.91), fibre length (*r* = 0.95), vessel length (*r* = 0.82) and vessel lumen area (*r* = 0.83). Species with high WUE and long vessels and fibres, such as *N. guianensis* and *A. discolor*, were positioned at one extreme of this axis while other species distribute along the other half of the axis. The second axis explained 19.3 % of the total variation. This axis was strongly correlated to leaf mass per area (*r* = 0.84) and leaf succulence (*r* = 0.66). Species showing greater leaf succulence and specific leaf mass, such as *S. versicolor*, *M. guianensis*, and *N. guianensis*, showed higher scores on this axis. The third axis and fourth axis of variation **[see Supporting Information—Figure S10]** together explained less than 15 % of the variation. The third axis is moderately correlated to WUE (*r* = 0.61) and K_h_ (*r* = −0.57) and the fourth axis is to WSG (*r* = −0.57). All other variables showed correlation coefficients that were below 0.5 **[see Supporting Information—Table S1]**. A total of 44 % of trait variation is explained by the species identity and trait variation among species is more than two times higher than within each species (PermANOVA, *F* = 5.4751, *R*^2^ = 0.66, *P* menor 0.005).

### Relationships between anatomical and hydraulic traits

**Figure 6.**
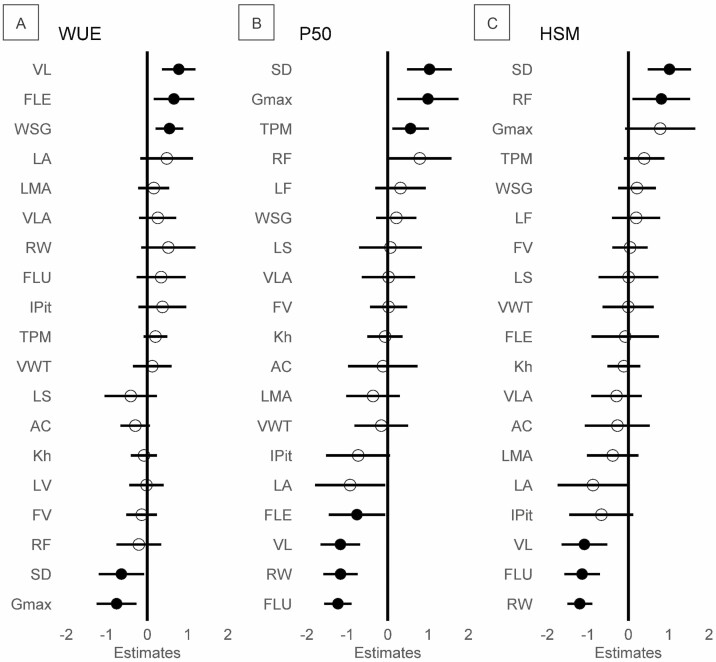
Effects of anatomical features on the hydraulic traits of (A) water-use efficiency, (B) drought-induced embolism resistance, and (C) hydraulic safety margin. Each line represents the final generalized mixed model for a given response variable after model selection. Standardized regression coefficients are plotted for each model associated with a 95% confidence interval. Coefficients different from zero at a significance level below 0.05 are shown in black. Note that variables are not presented in the same order in each plot but are sorted by effect size to aid visualization. LA: leaf area; LS: leaf succulence; LMA: leaf mass per area; SD: stomatal density; AC: adaxial cuticle; *G*_max_(Gmx): theoretical maximum stomatal conductance; δ¹³C: water-use efficiency; *K*_h_: theoretical hydraulic conductivity; VLA: vessel lumen area; FV: frequency vessel; VL: vessel element length; LF: lumen fraction; RF: ray frequency; RW: ray width; FLU: fibre lumen; FLE: fibre length; WSG: wood-specific gravity; VWT: vessel wall thickness; IPit: intervessel pits; TPM: thickness pit membrane; P_50_: embolism resistance; HSM: hydraulic safety.

Hydraulic traits were related to anatomical traits in 17 of the 57 bivariate relationships analyzed (Fig. 6, **[see Supporting Information—Table S3]**), with anatomical in leaf and stem being related to hydraulic traits in both organs. Stomata density and vessel element length were the only anatomical traits related to all three hydraulic traits. *G*_max_ and fibre length were related to both WUE and P_50_, while fibre lumen and ray width were related to P_50_ and HSM. All other anatomical traits were related to single or none hydraulic traits. WUE increased with vessel element length (slope: 0.78, *P* = 0.002), fibre length (slope: 0.66, *P* = 0.018), wood density (slope: 0.55, *P* = 0.006), decreased with stomata density (slope: −0.64, *P* = 0.036) and *G*_max_ (slope: −0.76, *P* = 0.008). In the contrast, P_50_ increased with *G*_max_ (slope: 0.99, *P* = 0.023), stomata density (slope: 1.03, *P* = 0.004), thickness pit membrane (slope: 0.56, *P* = 0.028), decreased with vessel element length (slope: −1.18, *P* = 0.001), fibre length (slope: −0.76, *P* = 0.047), but also with ray width (slope: −1.17, *P* < 0.001) and fibre lumen (slope: −1.24, *P* < 0.001). Finally, HSM increased with stomata density (slope: 1.02, *P* = 0.004) and ray frequency (slope: 0.82, *P* = 0.041) and decreased with vessel element length (slope: −1.09, *P* = 0.003), fibre lumen (slope: −1.14, *P* = 0.001) and ray width (slope: −1.20, *P* < 0.001).

## Discussion

Here, we show that few anatomical traits are related to hydraulic traits, even when many of them are related to each other. Our findings showed a wide variation in resistance to embolism, WUE and structural anatomy, suggesting no unique dominant functional strategy among Amazonian savanna species. There, species diverge by showing a different combination of WUE and drought-induced embolism resistance. Here, we found species with high *G*_max_ and low WUE that invest in leaf succulence to delay embolism onset (e.g. *Simarouba versicolor* and *Macairea radula*) or show thick intervascular pit membranes to minimize the embolism spread across the plant (e.g. *Maprounea guianensis* and *Parkia cachimboensis*) to control water loss. In contrast, we found that species with low G_max_ and high WUE show consistently longer element vessel lengths, greater vessel lumen areas with more limited lumen fraction, and longer fibre lengths with wider fibre lumen (e.g. *Norantea guianensis* and *Alchornea discolor*). Together, our results provide a deeper understanding of how branch and leaf structural traits combine to allow for different hydraulic strategies among coexisting plants, converging on safe structures, although in different organs.

### Xylem structure and functioning

Amazonian savanna species studied here diverged widely regarding their vulnerability to embolism. We observed extremely vulnerable species, such as *N. guianensis* (P_50_ = −1.6 MPa), co-existing with drought-resistant species, such as *P. cachimboensis* (−5.0 MPa), *M. guianensis* (−4.5 MPa), *S. versicolor* (−4.4 MPa), and *K. rubriflora* (−3.8 MPa). In Amazonian forest trees, P_50_ ranges between ~−0.5 and −5.0 (Rowland *et al*. 2015; Santiago *et al*. 2018; Barros *et al*. 2019; Bittencourt *et al*. 2020; Fontes *et al*. 2020). In comparison with previous studies, our results suggest that Amazonian savanna species, in general, are not more resistant to embolism than Amazonian forest species. This variation was observed when comparing safety margins among species, which also varied widely (Table 2). Although at the global scale is observed a very clear pattern of less negative P_50_ in wetter habitats and more negative P_50_ in drier habitats (Choat *et al*. 2012; O’Brien *et al*. 2017), similar amounts of variation can be also observed under the same climate among co-existing species as shown here.

Vessel characteristics directly determine water transport efficiency, and the hypothesis that there is a trade-off between the safety and efficiency of the xylem has often been raised (Gleason *et al*. 2016). This hypothesis proposes that the xylem anatomy drives safety and efficiency, but that the vessel characteristics cannot promote safety and efficiency at the same time. Our findings support this view as most resistant species do have shorter vessel elements with smaller diameters. The secondary xylem of this group of species is structured by a higher frequency of short vessel elements with thick pit membranes and smaller diameters, which safely increases the fraction of lumen for water transport. Flow resistance is augmented as element vessel size decreases as water needs to flow through a larger number of end walls with impacts on the plant’s hydraulic conductivity (Loepfe *et al*. 2007). Here, we show that plants with short vessel elements and higher frequency and clustering of vessels also show high lumen fraction. This may be associated with a strategy to compensate for the loss of hydraulic conductivity, as this hydraulic architecture increases the options for transport routes through the vessels.

### Hydraulic architecture and embolism resistance

Some controversy on the functional role of vessel grouping in xylem safety is present in the literature. Tyree and Zimmermann (2002) and Lens *et al*. (2011) showed that the grouping of vessels can improve embolism resistance. On the other hand, Loepfe *et al*. (2007) and Martínez-Vilalta *et al*. (2012) suggested the opposite, that is, high clustering of vessels is related to low security by increasing the probability of embolism propagation. Here, we observed greater clustering of vessels associated with embolism resistance confirming the importance of grouping to xylem safety. Again, in those species, protection against embolism spread may be guaranteed by thicker intervascular pit membranes, which are related to decreased air propagation from embolized vessels to functional vessels (Lens *et al*. 2011; Dória *et al*. 2018). Our results also confirm the functional importance of the intervascular pit membrane drought-induced embolism resistance in Amazonian savanna species. Here, we show that drought-induced embolism and HSM were significantly related to vessel element length and thickness of the membrane of the intervascular pit and that short vessel elements with thick membranes allow safe water transport for the studied species. Together, our results suggest that investigating the role of anatomical structures in hydraulic function in a more integrative way can reconcile the different expectations about the role of individual anatomical features in hydraulic functioning.

### Wood parenchyma variation

Finally, structural adjustments to allow safe or efficient water transport may not be restricted to the xylem but also be related to parenchymatic tissues. The limited space within the xylem and the resources available for its construction should lead to a three-way exchange among the main functions of the branch: mechanical support, conduction, and storage of water and photoassimilates (Pratt *et al*. 2007; Zanne *et al*. 2010). A study of 800 species of trees in China found axial parenchyma to be related to theoretical hydraulic conductivity, and radial parenchyma to be related to mechanical support (Zheng and Martínez-Cabrera 2013), which may explain the increase in radial parenchyma in species with greater lumen area. Chen *et al.* (2020) obtained similar results for 10 species in an arid limestone habitat in China. Their results showed that resistance to embolism was positively correlated with xylem mechanical resistance indicators and negatively related to the fraction of axial parenchyma. These authors suggested that tree species with more axial parenchyma may not need high-resistance to embolism to adapt to drought. The present study did not analyze the xylem at the parenchymatic tissue level, but variation in the amount of axial and radial parenchyma among species observed in the anatomical images suggests that this may be one of the mechanisms that allow vulnerable species (e.g. *Norantea guianensis*) to persist on Amazonian savanna’s environmental conditions. Future studies relating anatomical features, tissue capacitance, and xylem function may shed some light on this interesting strategy.

### Local divergence in leaf and branch traits

The main novelty of the present study is that it shows no convergence of traits for species to be dominant in Amazonian savannas, but rather there is a set of anatomical and hydraulic attributes that vary together, reflecting the specific responses of each species to drought. Our results demonstrate a strong degree of co-variation between water transport and stomatal regulation. Here, we show that species that are less efficient in water use (e.g. *K. rubriflora*, *M. radula*, *S. versicolor*, *P. cachimboensis* and *M. guianensis*) show higher stomatal conductance potential (high G_max_) supporting xylem functioning with leaf succulence and/or safer wood anatomical structures. Alternatively, species that are more efficient in water use (e.g. *N. guianensis* and *A. discolor*) can exhibit riskier hydraulic strategies. The coupling between low stomatal conductance and more negative xylem tension as we found here suggests integration between stomatal sensitivity and xylem vulnerability (Brodribb *et al*. 2003). In this case, a reduction in stomatal conductance should be accompanied by a decline in leaf photosynthesis and increased WUE, with greater stomatal control in the dry season (Janssen *et al*. 2020) as observed here. Vessel element length and pit membrane thickness explained resistance to embolism while leaf succulence allows for less conservative water use strategies. Allometry appears to be regulated at the scale of the entire plant so that water demand and supply are compatible (Brodribb 2009). This preliminary result suggests that Amazonian savannas plants combine trait variation within leaf and wood to sustain a positive water balance, but this hypothesis remains to be tested.

## Conclusion

This study provides new insights into the different structural and hydraulic traits that plants can exhibit and represents the first work to estimate xylem embolism resistance in Amazonian savanna species. Our results suggest that species that inhabit hot and seasonal savannas can exhibit different strategies of drought tolerance/avoidance. These strategies can be equally successful in the maintenance of a favourable water balance, allowing species persistence and co-occurrence. Distinct strategies to cope with drought permitted wide divergence regarding embolism vulnerability. However, the role of traits related to drought avoidance should not be neglected in this ecosystem. Future climate predictions should aim to incorporate this range of strategies and could be used to test whether future climates will favour some strategies or still allow for a range of them to persist. Further studies and refinement of model exercises are likely to improve our predictions on changing the species and trait composition of Amazonian savannas and also its impact on large-scale ecosystem functioning.

## Supporting Information

The following additional information is available in the online version of this article –

**Figure S1**. Examples of vessel element measurement for (left) *Alcornea discolor*, a species with a long vessel element and (right) *Parkia cachimboensis*, a species with short vessel element. Please note that the vessel element length was given by the maximum length of the feature, which included the vessel element tip.

**Figure S2**. Examples of intervessel pit size measurements for *Alchonea discolor*, *Kielmeyera rubriflora*, *Simarouba versicolor*, *Norantea guianensis* and *Parkia cachiboensis* evidencing the size variation in intervessel pit size among species. Pit size value is given by the average between 25 pit size measurements within the same individual.

**Figure S3**. Examples of intervessel element thickness measurement for (left) *Maprounea guianensis* and (right) *Macairea radula*. Please note that the cell wall thickness was individually measure in each one of the two walls.

**Figure S4**. Examples of pit membrane thickness measurements *Macairea radula*, *Alchonea discolor* and *Maprounea guianensis*. Please note that three measures were made per sample and the measurements averaged to provide a single value per sample.

**Figure S5**: Monthly temperature, relative humidity, and precipitation in the Amazon savanna (10º 53’98.7 ‘55º 46’68.7’) where this study samples were collected in the year 2019. The data are from a meteorological unit located in the area and were provided by the *Usina Hidrelétrica de Colider – Nova Canaã do Norte/Mato Grosso*, company responsible for this monitoring.

**Figure S6**: Xylem vulnerability curves. Relationship between percentage loss of conductivity and water potential. P50 represents the water potential corresponding to a 50% loss of xylem conductivity. Different colors represent different sampled individuals. Species: *Parkia cachimboensis*; *Maprounea guianensis*; *Simarouba versicolor*; *Kielmeyera rubriflora*; *Norantea guianensis*.

**Figure S7:** Effects of anatomical features on the hydraulic traits of (A) Water-Use Efficiency, (B) Drought-Induced Embolism resistance and (C) Hydraulic Safety Margin. Each line represents the **linear model** for a given response variable after model selection. Standardized regression coefficients are plotted for each model associated with a 95% confidence interval. Coefficients different from zero at a significance level below 0.05 are shown in black. Note that variables are not presented in the same order in each plot but sorted by effect size to aid visualization. LA-Leaf area; LS-Leaf succulence; LMA- Leaf mass per area; SD- Stomatal density; AC- Adaxial cuticle; Gmax (Gmx)- Theoretical maximum stomatal conductance; WUE (δ¹³C)- water use efficiency; Kh- theoretical hydraulic conductivity; VLA- Vessel lumen area; FV- Frequency vessel; VL- Vessel element length; LF-Lumen fraction; RF-Ray frequency; RW- Ray width; FLU- Fiber lumen; FLE- Fiber length; WSG- Wood specific gravity; VWT- Vessel wall thickness; IPit- Intervessel pits; TPM-Thickness pit membrane; P50- Embolism resistance; HSM- hydraulic safety.

**Figure S8**: Intraspecific and interspecific variation in morphological, anatomical and hydraulic traits of leaf (A; B; C; D; E) and wood (F; G; H; I; J), P_50_ (K) and HSM (L) for each of the sampled species. Ad - *Alchornea discolor*, Kr - *Kielmeyera rubriflora*; Mr - *Macairea radula*; Mg - *Maprounea guianensis*; Ng - *Norantea guianensis*; Pc - *Parkia cachimboensis*; Sv - *Simarouba versicolor*. *Species sampled with P_50_ and HSM measures.

**Figure S9:** Matrix of Spearman’s correlation coefficients between anatomical and hydraulic leaf and wood traits. Red represents negative correlations and blue represents positive correlations (significance level is p <0.05).

**Figure S10:** Principal component analysis (PCA) of anatomical and WUE (δ¹³C)- water use efficiency with species coded by colours. LA-Leaf area; LS-Leaf succulence; LMA- Leaf mass per area; SD- Stomatal density; AC- Adaxial cuticle; G_max_ - Theoretical maximum stomatal conductance; WUE (δ¹³C)- water use efficiency; Kh- theoretical hydraulic conductivity; VLA- Vessel lumen area; FV- Frequency vessel; VL- Vessel element length; LF-Lumen fraction; RF-Ray frequency; RW- Ray width; FLU- Fiber lumen; FLE- Fiber length; WSG- Wood specific gravity; VWT- Vessel wall thickness; IPit- Intervessel pits; TPM-Thickness pit membrane.

**Table S1**: Correlation coefficients between PCA loadings and anatomical and WUE (δ¹³C)- water use efficiency. Red tones denote medium to high positive correlations, green tones denote medium to high negative correlations and yellow tones week correlations. LA-Leaf area; LS-Leaf succulence; LMA- Leaf mass per area; SD- Stomatal density; AC- Adaxial cuticle; G_max_ - Theoretical maximum stomatal conductance; δ¹³C- water use efficiency; Kh- theoretical hydraulic conductivity; VLA- Vessel lumen area; FV- Frequency vessel; VL- Vessel element length; LF-Lumen fraction; RF-Ray frequency; RW- Ray width; FLU- Fiber lumen; FLE- Fiber length; WSG- Wood specific gravity; VWT- Vessel wall thickness; IPit- Intervessel pits; TPM-Thickness pit membrane.

**Table S2**: Results of the final generalized linear models for water use efficiency (WUE), drought-induced embolism resistance (P50) and hydraulic safety margin (HSM), including all anatomic traits. LA-Leaf area; LS-Leaf succulence; LMA- Leaf mass per area; SD- Stomatal density; AC- Adaxial cuticle; G_max_ - Theoretical maximum stomatal conductance; δ¹³C- water use efficiency; Kh- theoretical hydraulic conductivity; VLA- Vessel lumen area; FV- Frequency vessel; VL- Vessel element length; LF-Lumen fraction; RF-Ray frequency; RW- Ray width; FLU- Fiber lumen; FLE- Fiber length; WSG- Wood specific gravity; VWT- Vessel wall thickness; IPit- Intervessel pits; TPM-Thickness pit membrane.

**Table S3**: Results of the final generalized mixed models for water use efficiency (WUE), drought-induced embolism resistance (P50) and hydraulic safety margin (HSM), including all anatomic traits as fixed factor and species-nested-within-genus and genus-nested-within species as random intercepts. LA-Leaf area; LS-Leaf succulence; LMA- Leaf mass per area; SD- Stomatal density; AC- Adaxial cuticle; G_max_ - Theoretical maximum stomatal conductance; δ¹³C- water use efficiency; Kh- theoretical hydraulic conductivity; VLA- Vessel lumen area; FV- Frequency vessel; VL- Vessel element length; LF-Lumen fraction; RF-Ray frequency; RW- Ray width; FLU- Fiber lumen; FLE- Fiber length; WSG- Wood specific gravity; VWT- Vessel wall thickness; IPit- Intervessel pits; TPM-Thickness pit membrane.

plad018_suppl_Supplementary_Data_S1

plad018_suppl_Supplementary_Data_S2

plad018_suppl_Supplementary_Data_S3

plad018_suppl_Supplementary_Files

## List supporting data files supplied

1 data_base_Simioni *et al* 2023 - data table to run analysis.

2 code_analysis_Simoni *et al* 2023 - analysis script details.

3 data_pneumatic_method_Simioni *et al* 2023 - embolism resistance analysis data table.

4 Pneumatic_method_code_Simioni *et al* 2023 - embolism resistance analysis script details.

## Data Availability

The data that support the findings of this study are incorporated into the article and its Supporting Information. Further inquiries can be directed to the corresponding author.

## Notes

**Form & Function. Chief Editor:** Kate McCulloh
