# Supplementary material for: Anatomical traits related to leaf and branch hydraulic functioning on Amazonian savanna plants
Source: AoB Plants. 2023 Apr 24;15(3):plad018. doi: 10.1093/aobpla/plad018 (PMC10198777; doi:10.1093/aobpla/plad018)
Supplement: plad018_suppl_Supplementary_Data_S1 [file plad018_suppl_supplementary_data_s1.docx]

#### Anatomical traits related to leaf and branch hydraulic functioning on Amazonian savanna plants

library(qgraph)# trait correlation networks

library(vegan)#distance metrics

library(permute)#permANOVA

library(nlme)#glmm

library(FactoMineR) ##PCA

library (MuMIn) ### Calculate conditional and marginal coefficient of determination

dir()

# data prep

data<-read.csv("data.csv", stringsAsFactors = F)

head(data)

colnames(data)

#corData<- na.omit(data)

corDataAll<- data[,c(6:27)] # filtering characters column out

corDataAll<-na.omit(corDataAll) # filtering NA out to correlation for all traits ( anatomical and hydraulics traits)

corDataAn<-data[,c(6:10,14:25)] # filtering only anatomical traits

pcaData<-data[,5:25] # filtering species column character out excluding HSM and P50

pAnovaData<- data[,c(5:25)] # filtering only code sp and all traits excluding HSM and P50

# Spearman correlation network

##Fig 4

###A - only anatomic data

qgraph(cor(corDataAn), posCol="darkblue",negCol="darkred", graph="cor", minimum="sig", sampleSize=nrow(corDataAn))

####B - anatomic and hydraulic data

qgraph(cor(corDataAll), posCol="darkblue",negCol="darkred", graph="cor", minimum="sig", sampleSize=nrow(corDataAll))

#Principal component analysis (PCA)

## Analysis for Fig 5 and S10

pca <- PCA(pcaData[,-1], ncp= 22, graph = T, scale.unit=T)

summary(pca)

## Table S1

pca$var$cor

## Permanova

distance= vegdist(abs(pAnovaData[,-1]), method="euclidian", na.rm=T)

permanova=adonis2(formula = distance ~ codesp, permutations = 10000,data=pAnovaData, na.omit=T)

summary(permanova)

permanova

#Generalized linear mixed-effects random intercept models

## Fig 6 and Table S3 model using P50 and LA as an example

fit1.p50 = lme(P50~scale(LA), random = ~ 1|species/genus/family, data = data, na.action = na.omit, control = lmeControl(opt = 'optim'), method = "ML")

r.squaredGLMM(fit1.p50)

#Least-square models

## Fig S9 and Table S3 model using P50 and LA as an example

fit1.p50.lm = lm(P50~scale(LA), data = data, na.action = na.omit)

summary(fit1.p50.lm)
