# Supplementary material for: Anatomical traits related to leaf and branch hydraulic functioning on Amazonian savanna plants
Source: AoB Plants. 2023 Apr 24;15(3):plad018. doi: 10.1093/aobpla/plad018 (PMC10198777; doi:10.1093/aobpla/plad018)
Supplement: plad018_suppl_Supplementary_Files [file plad018_suppl_supplementary_files.docx]

**Anatomical traits related to leaf and branch hydraulic functioning on Amazonian savanna plants**

**Running title:** Anatomical and hydraulic traits in Amazonian savanna plants

Priscila F. Simioni¹, Thaise Emilio^5*^, André L. Giles², Gustavo Viana de Freitas³, Rafael Silva Oliveira², Lara Setime³, Ângela Pierre Vitoria³, Saulo Pireda³, Ivone Vieira da Silva^4^_,_ Maura Da Cunha^1,3^

^1^ Programa de Pós-Graduação em Ecologia e Recursos Naturais, Universidade Estadual do Norte Fluminense Darcy Ribeiro, Campos dos Goytacazes, RJ, Brasil.

^2^ Departamento de Biologia Vegetal, Instituto de Biologia, UNICAMP, Campinas, Brasil.

^3^ Laboratório de Biologia Celular e Tecidual, Universidade Estadual do Norte Fluminense Darcy Ribeiro, Campos dos Goytacazes, RJ, Brasil.

^4^ Laboratório de Biologia Vegetal, Universidade do Estado do Mato Grosso, Alta Floresta, MT, Brasil.

^5^ Programa Nacional de Pós-Doutorado (PNPD), Programa de Pós-Graduação em Ecologia, Instituto de Biologia, UNICAMP, Campinas, Brasil.


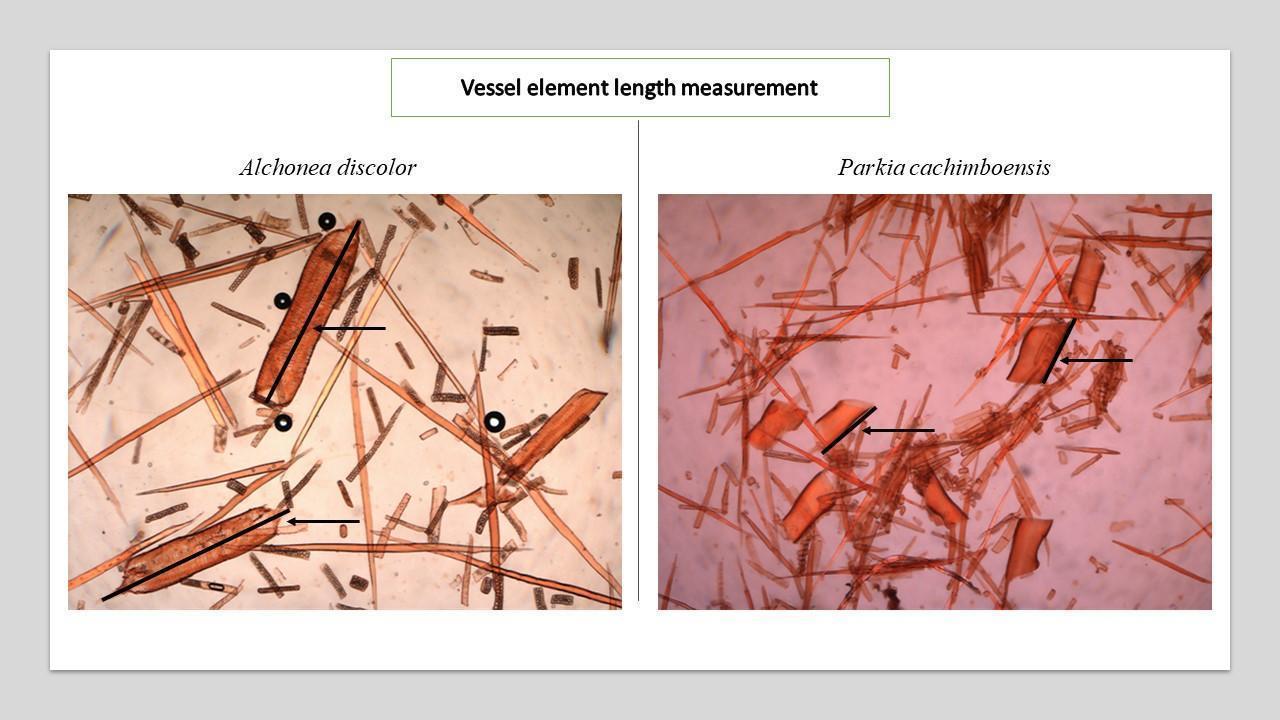


**Figure S1**. Examples of vessel element measurement for (left) *Alcornea discolor*, a species with a long vessel element and (right) *Parkia cachimboensis*, a species with short vessel element. Please note that the vessel element length was given by the maximum length of the feature, which included the vessel element tip.


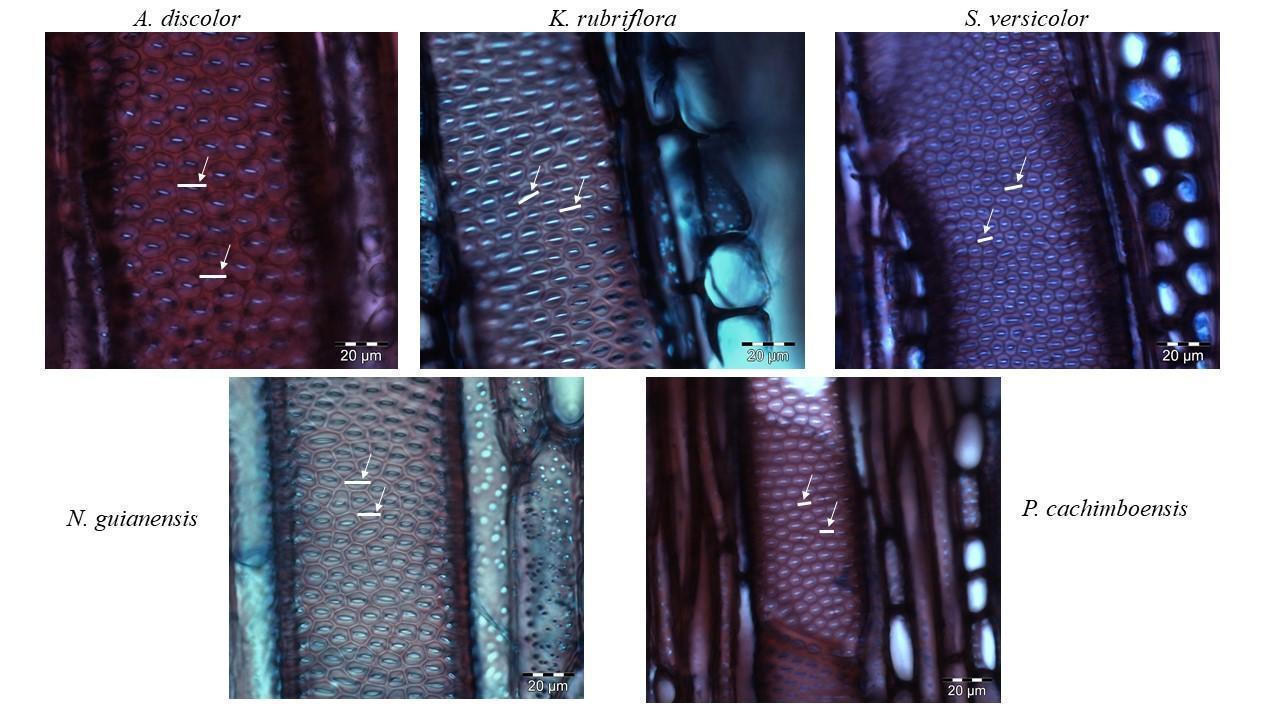


**Figure S2**. Examples of intervessel pit size measurements for *Alchonea discolor*, *Kielmeyera rubriflora*, *Simarouba versicolor*, *Norantea guianensis* and *Parkia cachiboensis* evidencing the size variation in intervessel pit size among species. Pit size value is given by the average between 25 pit size measurements within the same individual.


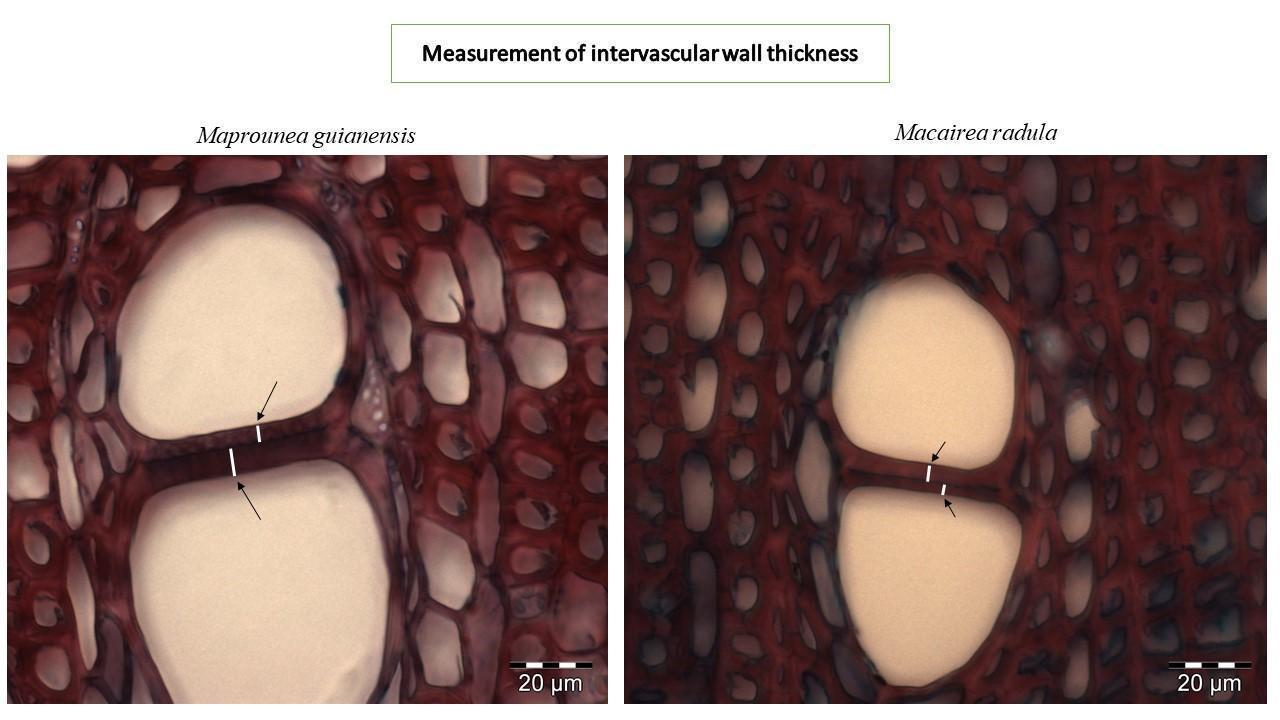


**Figure S3**. Examples of intervessel element thickness measurement for (left) *Maprounea guianensis* and (right) *Macairea radula*. Please note that the cell wall thickness was individually measure in each one of the two walls.


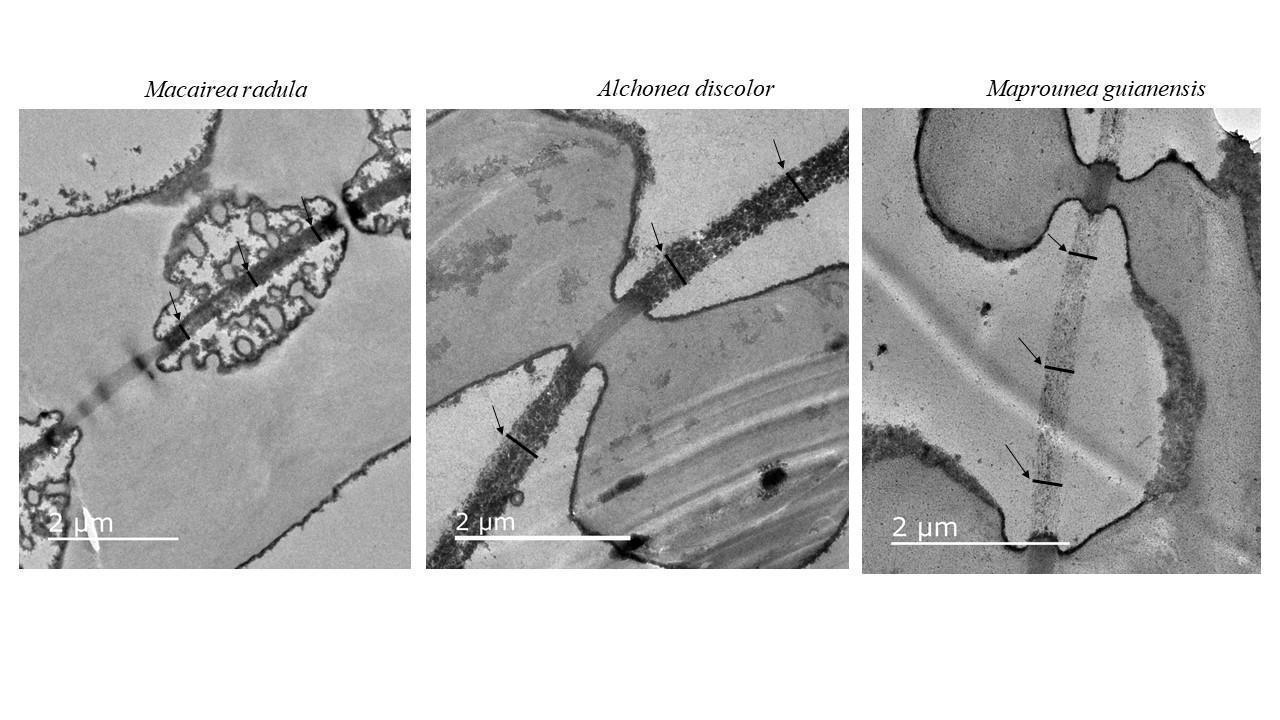


**Figure S4**. Examples of pit membrane thickness measurements *Macairea radula*, *Alchonea discolor* and *Maprounea guianensis*. Please note that three measures were made per sample and the measurements averaged to provide a single value per sample.


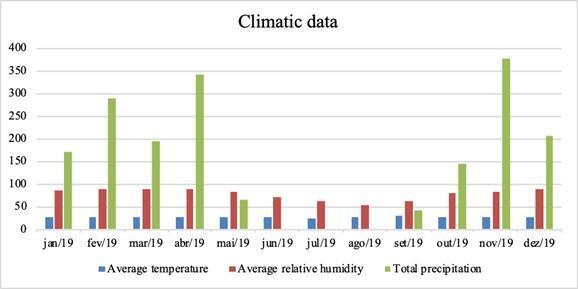


**Figure S5**: Monthly temperature, relative humidity, and precipitation in the Amazon savanna (10º 53'98.7 "55º 46'68.7") where this study samples were collected in the year 2019. The data are from a meteorological unit located in the area and were provided by the *Usina Hidrelétrica de Colider – Nova Canaã do Norte/Mato Grosso*, company responsible for this monitoring.


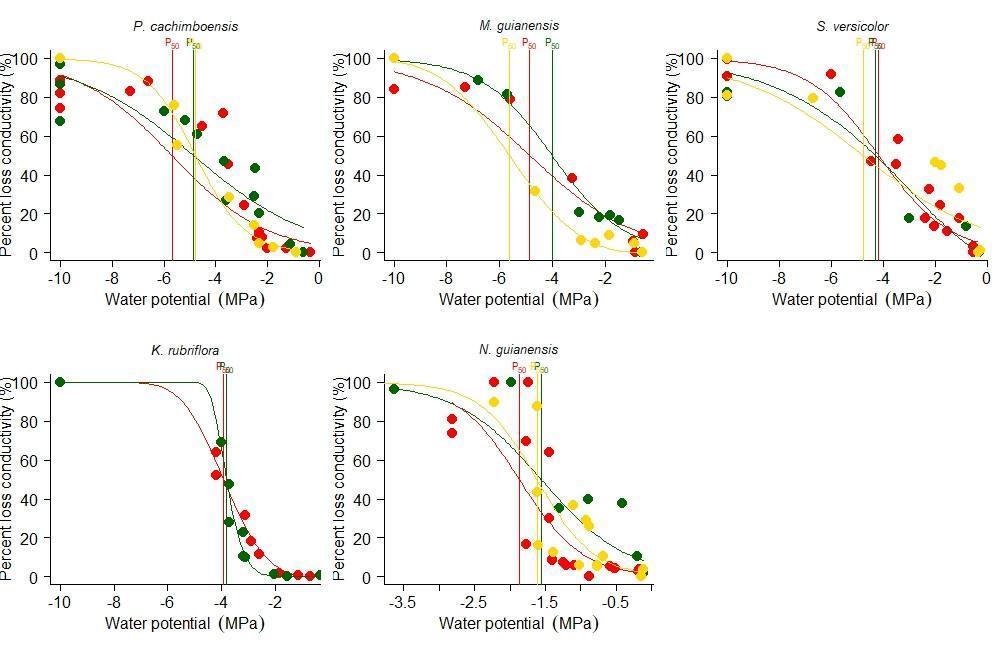


**Figure S6**: Xylem vulnerability curves. Relationship between percentage loss of conductivity and water potential. P50 represents the water potential corresponding to a 50% loss of xylem conductivity. Different colors represent different sampled individuals. Species: *Parkia cachimboensis*; *Maprounea guianensis*; *Simarouba versicolor*; *Kielmeyera rubriflora*; *Norantea guianensis*.

**
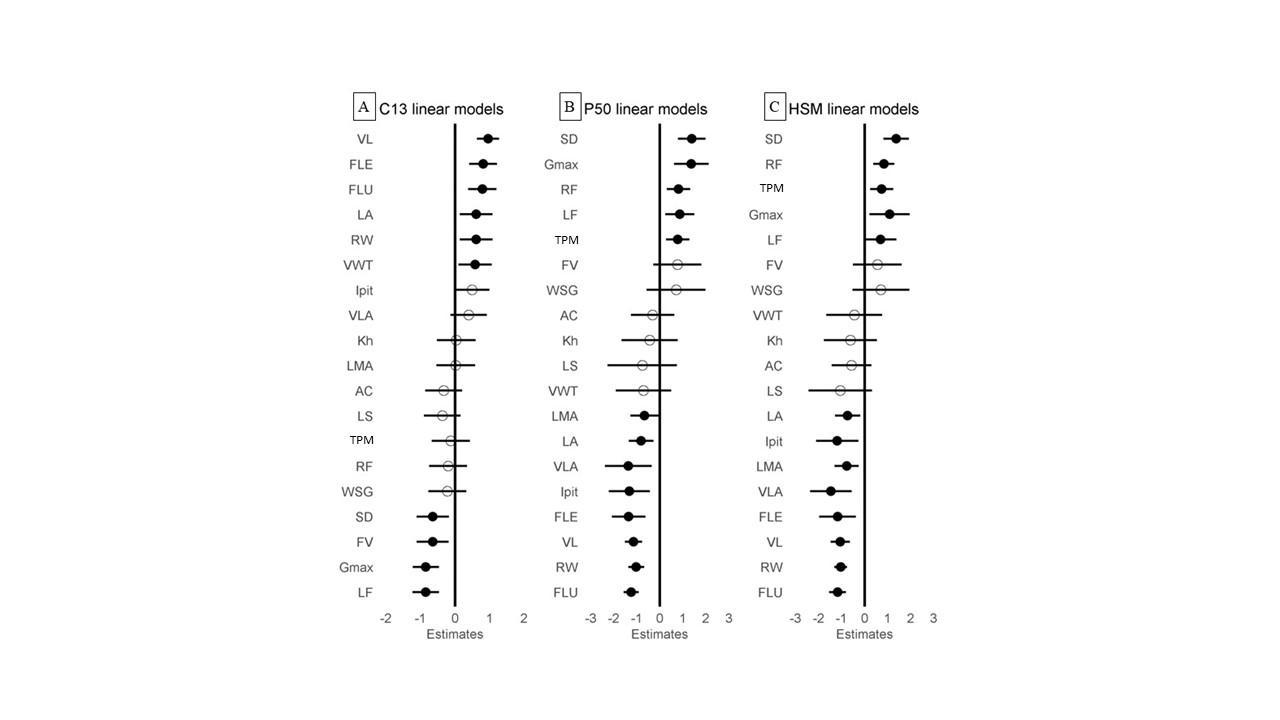
**

**Figure S7:** Effects of anatomical features on the hydraulic traits of (A) Water-Use Efficiency, (B) Drought-Induced Embolism resistance and (C) Hydraulic Safety Margin. Each line represents the **linear model** for a given response variable after model selection. Standardized regression coefficients are plotted for each model associated with a 95% confidence interval. Coefficients different from zero at a significance level below 0.05 are shown in black. Note that variables are not presented in the same order in each plot but sorted by effect size to aid visualization. LA-Leaf area; LS-Leaf succulence; LMA- Leaf mass per area; SD- Stomatal density; AC- Adaxial cuticle; Gmax (Gmx)- Theoretical maximum stomatal conductance; WUE (δ¹³C)- water use efficiency; Kh- theoretical hydraulic conductivity; VLA- Vessel lumen area; FV- Frequency vessel; VL- Vessel element length; LF -Lumen fraction; RF-Ray frequency; RW- Ray width; FLU- Fiber lumen; FLE- Fiber length; WSG- Wood specific gravity; VWT- Vessel wall thickness; IPit- Intervessel pits; TPM-Thickness pit membrane; P50- Embolism resistance; HSM- hydraulic safety.


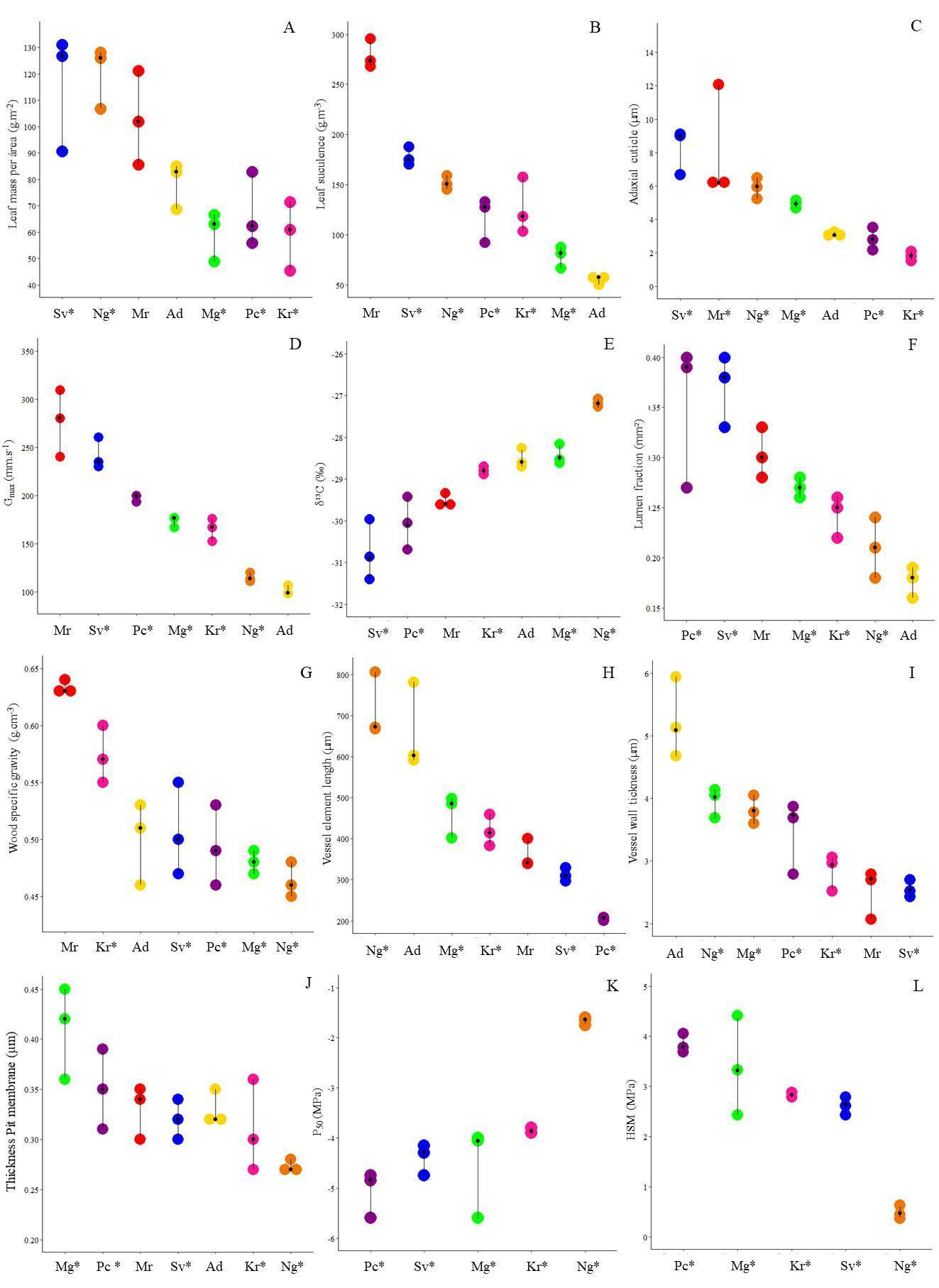


**Figure S8**: Intraspecific and interspecific variation in morphological, anatomical and hydraulic traits of leaf (A; B; C; D; E) and wood (F; G; H; I; J), P_50_ (K) and HSM (L) for each of the sampled species. Ad - *Alchornea discolor*, Kr - *Kielmeyera rubriflora*; Mr - *Macairea radula*; Mg - *Maprounea guianensis*; Ng - *Norantea guianensis*; Pc - *Parkia cachimboensis*; Sv - *Simarouba versicolor*. *Species sampled with P_50_ and HSM measures.

**
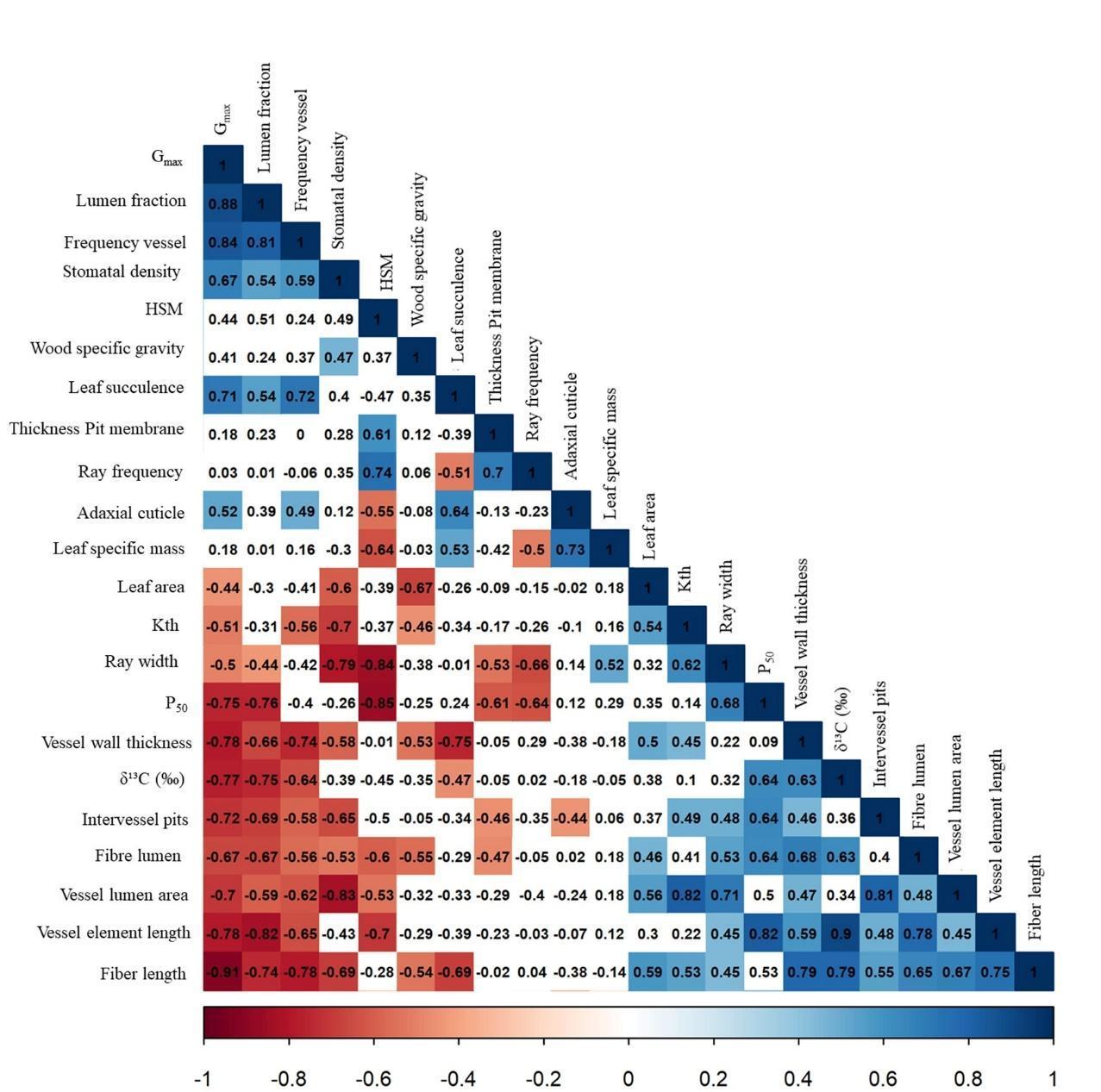
**

**Figure S9:** Matrix of Spearman's correlation coefficients between anatomical and hydraulic leaf and wood traits. Red represents negative correlations and blue represents positive correlations (significance level is p <0.05).


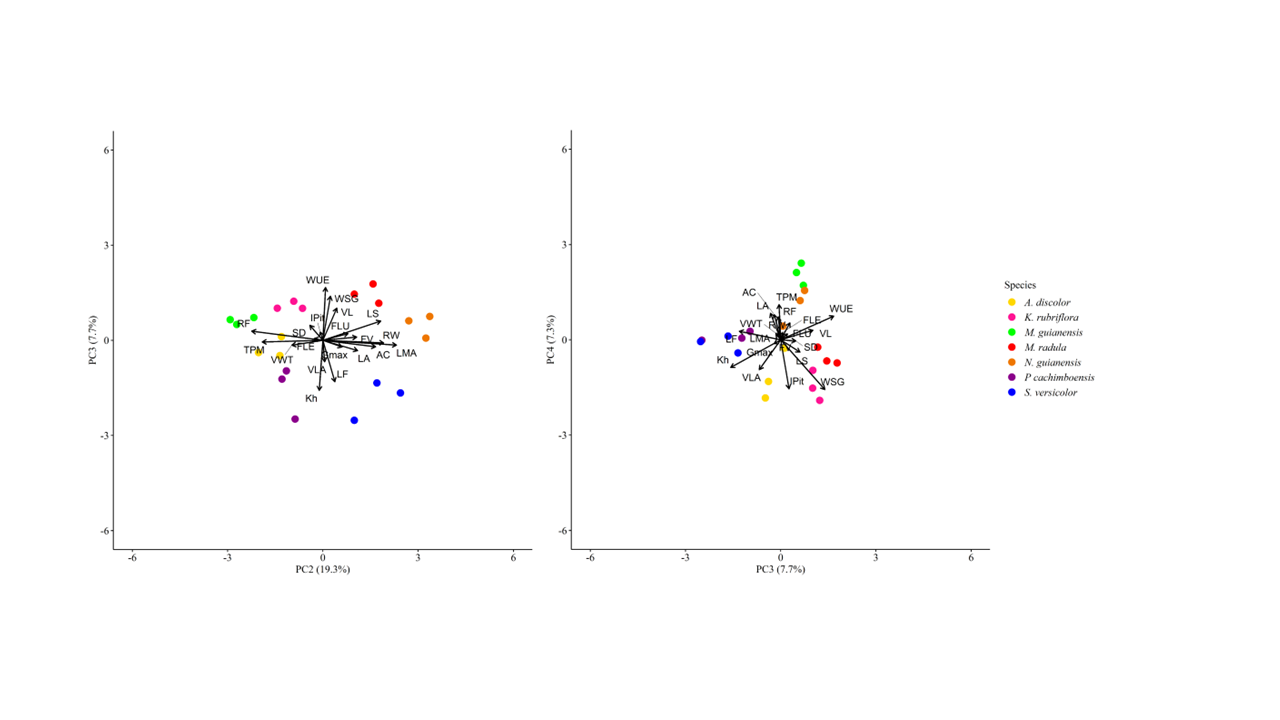


**Figure S10:** Principal component analysis (PCA) of anatomical and WUE (δ¹³C)- water use efficiency with species coded by colours. LA-Leaf area; LS-Leaf succulence; LMA- Leaf mass per area; SD- Stomatal density; AC- Adaxial cuticle; G_max_ - Theoretical maximum stomatal conductance; WUE (δ¹³C)- water use efficiency; Kh- theoretical hydraulic conductivity; VLA- Vessel lumen area; FV- Frequency vessel; VL- Vessel element length; LF -Lumen fraction; RF-Ray frequency; RW- Ray width; FLU- Fiber lumen; FLE- Fiber length; WSG- Wood specific gravity; VWT- Vessel wall thickness; IPit- Intervessel pits; TPM-Thickness pit membrane.


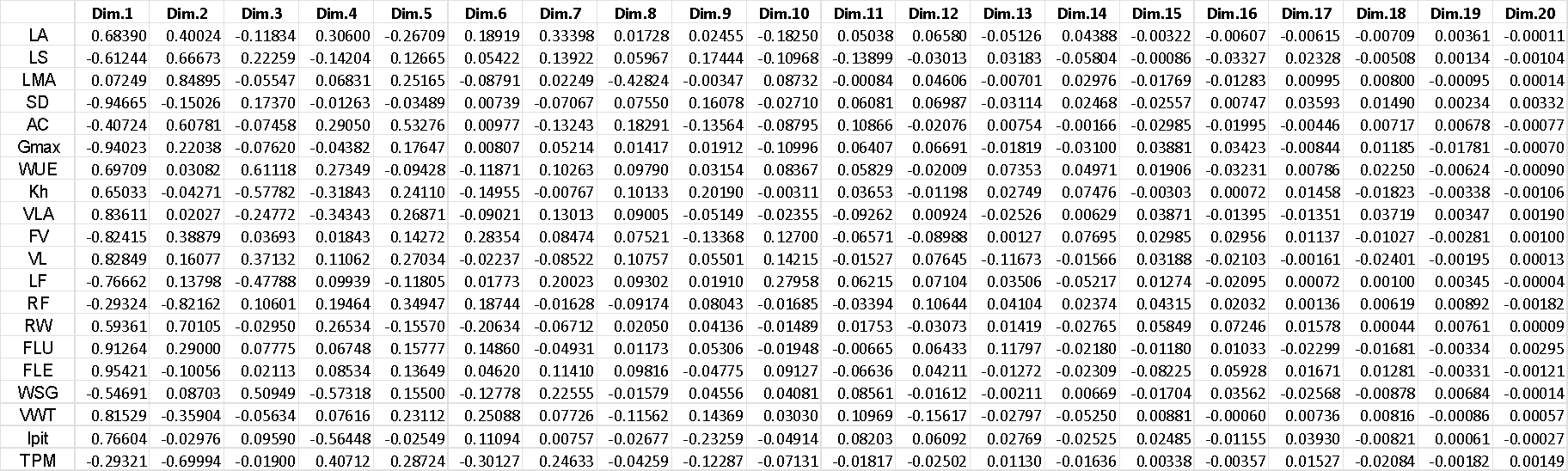


Table S1: Correlation coefficients between PCA loadings and anatomical and WUE (δ¹³C)- water use efficiency. Red tones denote medium to high positive correlations, green tones denote medium to high negative correlations and yellow tones week correlations. LA-Leaf area; LS-Leaf succulence; LMA- Leaf mass per area; SD- Stomatal density; AC- Adaxial cuticle; G_max_ - Theoretical maximum stomatal conductance; δ¹³C- water use efficiency; Kh- theoretical hydraulic conductivity; VLA- Vessel lumen area; FV- Frequency vessel; VL- Vessel element length; LF -Lumen fraction; RF-Ray frequency; RW- Ray width; FLU- Fiber lumen; FLE- Fiber length; WSG- Wood specific gravity; VWT- Vessel wall thickness; IPit- Intervessel pits; TPM-Thickness pit membrane.

Table S3: Results of the final generalized mixed models for water use efficiency (WUE), drought-induced embolism resistance (P50) and hydraulic safety margin (HSM), including all anatomic traits as fixed factor and species-nested-within-genus and genus-nested-within species as random intercepts. LA-Leaf area; LS-Leaf succulence; LMA- Leaf mass per area; SD- Stomatal density; AC- Adaxial cuticle; G_max_ - Theoretical maximum stomatal conductance; δ¹³C- water use efficiency; Kh- theoretical hydraulic conductivity; VLA- Vessel lumen area; FV- Frequency vessel; VL- Vessel element length; LF-Lumen fraction; RF-Ray frequency; RW- Ray width; FLU- Fiber lumen; FLE- Fiber length; WSG- Wood specific gravity; VWT- Vessel wall thickness; IPit- Intervessel pits; TPM-Thickness pit membrane.

|  | **(Intercept)** | | |  |  | | | **R²m/**  **R²C** |
| --- | --- | --- | --- | --- | --- | --- | --- | --- |
|  | *Estimates* | *CI* | *p* |  | *Estimates* | *CI* | *p* |  |
| **WUE (δ¹³C)** | -29.04 | -29.79 – -28.29 | **<0.001** | LA | 0.48 | -0.18 – 1.14 | 0.156 | 0.195/0.864 |
|  | -29.04 | -29.89 – -28.20 | **<0.001** | LS | -0.41 | -1.06 – 0.24 | 0.220 | 0.126/0.893 |
|  | -29.04 | -29.94 – -28.14 | **<0.001** | LMA | 0.16 | -0.23 – 0.55 | 0.413 | 0.019/0.893 |
|  | -29.04 | -29.78 – -28.31 | **<0.001** | SD | -0.64 | -1.21 – -0.08 | **0.036** | 0.315/0.902 |
|  | -29.04 | -29.90 – -28.19 | **<0.001** | AC | -0.30 | -0.66 – 0.06 | 0.114 | 0.070/0.899 |
|  | -29.04 | -29.63 – -28.45 | **<0.001** | G_max_ | -0.76 | -1.26 – -0.26 | **0.008** | 0.486/0.889 |
|  | -29.04 | -29.94 – -28.15 | **<0.001** | K_h_ | -0.08 | -0.41 – 0.24 | 0.609 | 0.005/0.889 |
|  | -29.04 | -29.88 – -28.20 | **<0.001** | VLA | 0.26 | -0.20 – 0.72 | 0.270 | 0.054/0.881 |
|  | -29.04 | -29.87 – -28.21 | **<0.001** | FV | -0.14 | -0.52 – 0.24 | 0.464 | 0.016/0.864 |
|  | -29.04 | -29.52 – -28.57 | **<0.001** | VL | 0.78 | 0.36 – 1.20 | **0.002** | 0.575/0.843 |
|  | -29.04 | -29.92 – -28.17 | **<0.001** | LF | -0.02 | -0.45 – 0.41 | 0.921 | 0.003/0.877 |
|  | -29.04 | -29.92 – -28.17 | **<0.001** | RF | -0.21 | -0.77 – 0.35 | 0.453 | 0.033/0.886 |
|  | -29.04 | -29.79 – -28.29 | **<0.001** | RW | 0.52 | -0.15 – 1.20 | 0.137 | 0.223/0.873 |
|  | -29.04 | -29.76 – -28.33 | **<0.001** | FLU | 0.34 | -0.27 – 0.96 | 0.270 | 0.117/0.816 |
|  | -29.04 | -29.67 – -28.42 | **<0.001** | FLE | 0.66 | 0.16 – 1.16 | **0.018** | 0.389/0.873 |
|  | -29.04 | -30.13 – -27.96 | **<0.001** | WSG | 0.55 | 0.20 – 0.89 | **0.006** | 0.142/0.964 |
|  | -29.04 | -29.88 – -28.20 | **<0.001** | VWT | 0.12 | -0.36 – 0.60 | 0.611 | 0.012/0.866 |
|  | -29.04 | -29.85 – -28.24 | **<0.001** | Ipit | 0.38 | -0.22 – 0.97 | 0.217 | 0.115/0.878 |
|  | -29.04 | -29.97 – -28.11 | **<0.001** | TPM | 0.20 | -0.09 – 0.50 | 0.182 | 0.030/0.910 |
| **P50** | 3.87 | 2.98 – 4.76 | **<0.001** | LA | -0.94 | -1.81 – -0.07 | 0.051 | 0.499/  0.873 |
|  | 3.91 | 2.66 – 5.17 | **<0.001** | LS | 0.06 | -0.71 – 0.84 | 0.865 | 0.002/0.848 |
|  | 3.89 | 2.83 – 4.96 | **<0.001** | LMA | -0.37 | -1.04 – 0.30 | 0.274 | 0.009/0.811 |
|  | 3.90 | 3.35 – 4.44 | **<0.001** | SD | 1.03 | 0.47 – 1.59 | **0.004** | 0.499/0.873 |
|  | 3.90 | 2.69 – 5.12 | **<0.001** | AC | -0.12 | -0.99 – 0.74 | 0.766 | 0.009/0.835 |
|  | 3.94 | 3.16 – 4.71 | **<0.001** | G_max_ | 0.99 | 0.24 – 1.75 | **0.023** | 0.581/0.870 |
|  | 3.91 | 2.69 – 5.13 | **<0.001** | K_h_ | -0.07 | -0.51 – 0.37 | 0.742 | 0.003/0.836 |
|  | 3.91 | 2.67 – 5.16 | **<0.001** | VLA | 0.02 | -0.64 – 0.67 | 0.961 | 0.001/0.840 |
|  | 3.91 | 2.69 – 5.14 | **<0.001** | FV | 0.02 | -0.45 – 0.48 | 0.934 | 0.001/0.833 |
|  | 3.91 | 3.42 – 4.40 | **<0.001** | VL | -1.18 | -1.67 – -0.68 | **0.001** | 0.799/0.889 |
|  | 3.93 | 2.88 – 4.97 | **<0.001** | LF | 0.31 | -0.31 – 0.94 | 0.318 | 0.075/0.789 |
|  | 3.92 | 3.08 – 4.75 | **<0.001** | RF | 0.79 | -0.00 – 1.58 | 0.065 | 0.695 |
|  | 3.91 | 3.50 – 4.32 | **<0.001** | RW | -1.17 | -1.60 – -0.75 | **<0.001** | 0.427/0.812 |
|  | 3.91 | 3.58 – 4.24 | **<0.001** | FLU | -1.24 | -1.58 – -0.89 | **<0.001** | 0.786/0.808 |
|  | 3.91 | 3.19 – 4.62 | **<0.001** | FLE | -0.76 | -1.46 – -0.07 | **0.047** | 0.433/0.668 |
|  | 3.89 | 2.70 – 5.08 | **<0.001** | WSG | 0.21 | -0.29 – 0.71 | 0.391 | 0.029/0.841 |
|  | 3.90 | 2.72 – 5.09 | **<0.001** | VWT | -0.16 | -0.83 – 0.50 | 0.616 | 0.017/0.829 |
|  | 3.95 | 3.06 – 4.85 | **<0.001** | Ipit | -0.73 | -1.53 – 0.06 | 0.083 | 0.372/0.822 |
|  | 3.92 | 2.98 – 4.87 | **<0.001** | TPM | 0.56 | 0.11 – 1.01 | **0.028** | 0.250/0.868 |
| **HSM** | 2.59 | 1.68 – 3.50 | **<0.001** | LA | -0.88 | -1.76 – 0.00 | 0.066 | 0.461/0.882 |
|  | 2.63 | 1.43 – 3.83 | **0.002** | LS | 0.00 | -0.74 – 0.75 | 0.994 | 0.000/0.848 |
|  | 2.61 | 1.65 – 3.58 | **<0.001** | LMA | -0.39 | -1.03 – 0.25 | 0.229 | 0.128/0.789 |
|  | 2.60 | 2.08 – 3.13 | **<0.001** | SD | 1.02 | 0.48 – 1.56 | **0.004** | 0.689/0.784 |
|  | 2.61 | 1.49 – 3.73 | **0.001** | AC | -0.27 | -1.08 – 0.53 | 0.491 | 0.461/0.880 |
|  | 2.65 | 1.71 – 3.59 | **<0.001** | G_max_ | 0.79 | -0.08 – 1.65 | 0.089 | 0.393/0.870 |
|  | 2.63 | 1.46 – 3.80 | **0.001** | K_h_ | -0.11 | -0.52 – 0.30 | 0.571 | 0.054/0.844 |
|  | 2.63 | 1.63 – 3.63 | **0.001** | VLA | -0.29 | -0.92 – 0.34 | 0.348 | 0.072/0.782 |
|  | 2.63 | 1.44 – 3.82 | **0.001** | FV | 0.04 | -0.40 – 0.48 | 0.844 | 0.008/0.844 |
|  | 2.63 | 2.04 – 3.22 | **<0.001** | VL | -1.09 | -1.66 – -0.52 | **0.003** | 0.732/0.901 |
|  | 2.64 | 1.53 – 3.75 | **0.001** | LF | 0.19 | -0.41 – 0.79 | 0.514 | 0.027/0.823 |
|  | 2.63 | 1.89 – 3.37 | **<0.001** | RF | 0.82 | 0.10 – 1.53 | **0.041** | 0.492/0.800 |
|  | 2.62 | 2.32 – 2.92 | **<0.001** | RW | -1.20 | -1.51 – -0.89 | **<0.001** | 0.860/0.900 |
|  | 2.61 | 2.18 – 3.03 | **<0.001** | FLU | -1.14 | -1.59 – -0.70 | **0.001** | 0.807/0.864 |
|  | 2.63 | 1.49 – 3.77 | **0.001** | FLE | -0.08 | -0.92 – 0.76 | 0.842 | 0.026/0.827 |
|  | 2.61 | 1.46 – 3.76 | **0.001** | WSG | 0.21 | -0.26 – 0.68 | 0.363 | 0.031/0.849 |
|  | 2.63 | 1.43 – 3.83 | **0.002** | VWT | -0.00 | -0.64 – 0.63 | 0.991 | 0.000/0.084 |
|  | 2.67 | 1.73 – 3.61 | **<0.001** | Ipit | -0.67 | -1.47 – 0.13 | 0.110 | 0.317/0.890 |
|  | 2.64 | 1.66 – 3.62 | **<0.001** | TPM | 0.39 | -0.11 – 0.89 | 0.138 | 0.317/0.849 |

Table S2: Results of the final generalized linear models for water use efficiency (WUE), drought-induced embolism resistance (P50) and hydraulic safety margin (HSM), including all anatomic traits. LA-Leaf area; LS-Leaf succulence; LMA- Leaf mass per area; SD- Stomatal density; AC- Adaxial cuticle; G_max_ - Theoretical maximum stomatal conductance; δ¹³C- water use efficiency; Kh- theoretical hydraulic conductivity; VLA- Vessel lumen area; FV- Frequency vessel; VL- Vessel element length; LF -Lumen fraction; RF-Ray frequency; RW- Ray width; FLU- Fiber lumen; FLE- Fiber length; WSG- Wood specific gravity; VWT- Vessel wall thickness; IPit- Intervessel pits; TPM-Thickness pit membrane.

|  | **(Intercept)** | | |  |  | | | **R²/ R²** **adjusted** |
| --- | --- | --- | --- | --- | --- | --- | --- | --- |
|  | *Estimates* | *CI* | *p* |  | *Estimates* | *CI* | *p* |  |
| **WUE (δ¹³C)** | -29.04 | -29.51 – -28.58 | **<0.001** | LA | 0.61 | 0.13 – 1.09 | **0.015** | 0.275/0.237 |
|  | -29.04 | -29.56 – -28.53 | **<0.001** | LS | -0.37 | -0.90 – 0.16 | 0.160 | 0.101/0.054 |
|  | -29.04 | -29.59 – -28.50 | **<0.001** | LMA | 0.02 | -0.54 – 0.58 | 0.945 | 0.000/-0.052 |
|  | -29.04 | -29.50 – -28.59 | **<0.001** | SD | -0.65 | -1.11 – -0.18 | **0.009** | 0.309/0.272 |
|  | -29.04 | -29.57 – -28.52 | **<0.001** | AC | -0.33 | -0.86 – 0.21 | 0.214 | 0.080/0.031 |
|  | -29.04 | -29.42 – -28.67 | **<0.001** | G_max_ | -0.85 | -1.23 – -0.46 | **<0.001** | 0.530/0.505 |
|  | -29.04 | -29.59 – -28.50 | **<0.001** | K_h_ | 0.03 | -0.52 – 0.59 | 0.898 | 0.001/-0.052 |
|  | -29.04 | -29.56 – -28.53 | **<0.001** | VLA | 0.39 | -0.13 – 0.92 | 0.135 | 0.113/0.067 |
|  | -29.04 | -29.50 – -28.59 | **<0.001** | FV | -0.65 | -1.11 – -0.19 | **0.009** | 0.312/0.275 |
|  | -29.04 | -29.35 – -28.73 | **<0.001** | VL | 0.96 | 0.64 – 1.27 | **<0.001** | 0.674/0.657 |
|  | -29.04 | -29.42 – -28.67 | **<0.001** | LF | -0.85 | -1.23 – -0.47 | **<0.001** | 0.532/0.507 |
|  | -29.04 | -29.58 – -28.51 | **<0.001** | RF | -0.20 | -0.75 – 0.35 | 0.445 | 0.031/-0.020 |
|  | -29.04 | -29.51 – -28.58 | **<0.001** | RW | 0.61 | 0.13 – 1.09 | **0.015** | 0.275/0.237 |
|  | -29.04 | -29.44 – -28.64 | **<0.001** | FLU | 0.79 | 0.38 – 1.20 | **0.001** | 0.462/0.433 |
|  | -29.04 | -29.43 – -28.65 | **<0.001** | FLE | 0.81 | 0.41 – 1.21 | **<0.001** | 0.487/0.460 |
|  | -29.04 | -29.58 – -28.51 | **<0.001** | WSG | -0.22 | -0.77 – 0.32 | 0.401 | 0.037/-0.013 |
|  | -29.04 | -29.51 – -28.57 | **<0.001** | VWT | 0.58 | 0.10 – 1.07 | **0.021** | 0.251/0.212 |
|  | -29.04 | -29.54 – -28.55 | **<0.001** | Ipit | 0.49 | -0.02 – 0.99 | 0.059 | 0.175/0.132 |
|  | -29.04 | -29.58 – -28.50 | **<0.001** | TPM | -0.12 | -0.68 – 0.43 | 0.644 | 0.011/-0.041 |
| **P50** | 4.06 | 3.46 – 4.66 | **<0.001** | LA | -0.81 | -1.35 – -0.27 | **0.007** | 0.474/0.430 |
|  | 3.82 | 3.03 – 4.62 | **<0.001** | LS | -0.76 | -2.27 – 0.74 | 0.290 | 0.092/0.017 |
|  | 3.91 | 3.24 – 4.59 | **<0.001** | LMA | -0.67 | -1.28 – -0.05 | **0.035** | 0.319/0.263 |
|  | 3.92 | 3.46 – 4.38 | **<0.001** | SD | 1.39 | 0.79 – 1.99 | **<0.001** | 0.679/0.652 |
|  | 3.91 | 3.11 – 4.71 | **<0.001** | AC | -0.32 | -1.26 – 0.62 | 0.476 | 0.043/-0.037 |
|  | 3.98 | 3.44 – 4.52 | **<0.001** | G_max_ | 1.37 | 0.62 – 2.13 | **0.002** | 0.566/0.530 |
|  | 3.91 | 3.11 – 4.70 | **<0.001** | K_h_ | -0.44 | -1.67 – 0.78 | 0.447 | 0.049/-0.030 |
|  | 3.70 | 3.05 – 4.34 | **<0.001** | VLA | -1.37 | -2.39 – -0.36 | **0.012** | 0.420/0.371 |
|  | 3.96 | 3.22 – 4.71 | **<0.001** | FV | 0.76 | -0.28 – 1.81 | 0.138 | 0.174/0.105 |
|  | 3.76 | 3.39 – 4.14 | **<0.001** | VL | -1.15 | -1.52 – -0.78 | **<0.001** | 0.791/0.773 |
|  | 3.73 | 3.09 – 4.36 | **<0.001** | LF | 0.87 | 0.23 – 1.50 | **0.012** | 0.425/0.377 |
|  | 4.02 | 3.44 – 4.60 | **<0.001** | RF | 0.81 | 0.30 – 1.32 | **0.005** | 0.499/0.458 |
|  | 4.15 | 3.76 – 4.54 | **<0.001** | RW | -1.03 | -1.37 – -0.68 | **<0.001** | 0.779/0.761 |
|  | 3.81 | 3.49 – 4.13 | **<0.001** | FLU | -1.24 | -1.57 – -0.91 | **<0.001** | 0.848/0.836 |
|  | 3.86 | 3.33 – 4.39 | **<0.001** | FLE | -1.35 | -2.08 – -0.62 | **0.002** | 0.575/0.540 |
|  | 4.20 | 3.27 – 5.13 | **<0.001** | WSG | 0.71 | -0.57 – 1.99 | 0.252 | 0.108/0.033 |
|  | 3.82 | 3.03 – 4.60 | **<0.001** | VWT | -0.71 | -1.92 – 0.49 | 0.222 | 0.122/0.048 |
|  | 3.95 | 3.06 – 4.85 | **<0.001** | Ipit | -0.73 | -1.53 – 0.06 | 0.083 | 0.677/NA |
|  | 3.87 | 3.28 – 4.45 | **<0.001** | TPM | 0.78 | 0.27 – 1.29 | **0.006** | 0.482/0.439 |
| **HSM** | 2.75 | 2.13 – 3.36 | **<0.001** | LA | -0.74 | -1.29 – -0.19 | **0.013** | 0.415/0.366 |
|  | 2.49 | 1.76 – 3.22 | **<0.001** | LS | -1.06 | -2.44 – 0.32 | 0.121 | 0.188/0.120 |
|  | 2.61 | 2.03 – 3.19 | **<0.001** | LMA | -0.78 | -1.31 – -0.25 | **0.007** | 0.463/0.418 |
|  | 2.62 | 2.19 – 3.05 | **<0.001** | SD | 1.37 | 0.82 – 1.93 | **<0.001** | 0.708/0.683 |
|  | 2.61 | 1.88 – 3.34 | **0.001** | AC | -0.57 | -1.43 – 0.30 | 0.178 | 0.145/0.074 |
|  | 2.67 | 2.04 – 3.29 | **<0.001** | G_max_ | 1.09 | 0.21 – 1.96 | **0.019** | 0.379/0.327 |
|  | 2.61 | 1.85 – 3.36 | **0.001** | K_h_ | -0.62 | -1.77 – 0.54 | 0.267 | 0.101/0.027 |
|  | 2.38 | 1.81 – 2.96 | **0.001** | VLA | -1.46 | -2.37 – -0.56 | **0.004** | 0.508/0.467 |
|  | 2.65 | 1.90 – 3.41 | **0.001** | FV | 0.56 | -0.50 – 1.61 | 0.275 | 0.098/0.023 |
|  | 2.48 | 2.05 – 2.90 | **<0.001** | VL | -1.06 | -1.48 – -0.64 | **<0.001** | 0.719/0.696 |
|  | 2.47 | 1.78 – 3.15 | **0.001** | LF | 0.69 | 0.01 – 1.38 | **0.047** | 0.289/0.230 |
|  | 2.73 | 2.20 – 3.25 | **<0.001** | RF | 0.84 | 0.38 – 1.30 | **0.002** | 0.572/0.537 |
|  | 2.86 | 2.55 – 3.17 | **<0.001** | RW | -1.04 | -1.31 – -0.77 | **<0.001** | 0.851/0.839 |
|  | 2.52 | 2.17 – 2.87 | **<0.001** | FLU | -1.17 | -1.54 – -0.81 | **<0.001** | 0.803/0.786 |
|  | 2.57 | 1.99 – 3.15 | **<0.001** | FLE | -1.17 | -1.97 – -0.38 | **0.008** | 0.461/0.416 |
|  | 2.91 | 2.01 – 3.81 | **<0.001** | WSG | 0.71 | -0.53 – 1.95 | 0.235 | 0.115/0.041 |
|  | 2.55 | 1.76 – 3.34 | **<0.001** | VWT | -0.45 | -1.66 – 0.76 | 0.434 | 0.052/-0.027 |
|  | 2.24 | 1.56 – 2.92 | **<0.001** | Ipit | -1.19 | -2.11 – -0.27 | **0.016** | 0.397/0.347 |
|  | 2.57 | 1.99 – 3.15 | **<0.001** | TPM | 0.74 | 0.24 – 1.25 | **0.007** | 0.465/0.421 |
